# Sex Hormones, Sleep, and Memory: Interrelationships Across the Adult Female Lifespan

**DOI:** 10.3389/fnagi.2022.800278

**Published:** 2022-07-14

**Authors:** Yasmin A. Harrington, Jeanine M. Parisi, Daisy Duan, Darlynn M. Rojo-Wissar, Calliope Holingue, Adam P. Spira

**Affiliations:** ^1^Department of Mental Health, Johns Hopkins Bloomberg School of Public Health, Baltimore, MD, United States; ^2^Division of Endocrinology, Diabetes and Metabolism, Johns Hopkins University School of Medicine, Baltimore, MD, United States; ^3^The Initiative on Stress, Trauma, and Resilience (STAR), Department of Psychiatry and Human Behavior, Center for Behavioral and Preventive Medicine, The Miriam Hospital, Warren Alpert Medical School of Brown University, Providence, RI, United States; ^4^Center for Autism and Related Disorders, Kennedy Krieger Institute, Baltimore, MD, United States; ^5^Department of Psychiatry and Behavioral Sciences, Johns Hopkins School of Medicine, Baltimore, MD, United States; ^6^Johns Hopkins Center on Aging and Health, Baltimore, MD, United States

**Keywords:** sleep, memory, sex, hormones, adults, dementia, Alzheimer’s disease

## Abstract

As the population of older adults grows, so will the prevalence of aging-related conditions, including memory impairments and sleep disturbances, both of which are more common among women. Compared to older men, older women are up to twice as likely to experience sleep disturbances and are at a higher risk of cognitive decline and Alzheimer’s disease and related dementias (ADRD). These sex differences may be attributed in part to fluctuations in levels of female sex hormones (i.e., estrogen and progesterone) that occur across the adult female lifespan. Though women tend to experience the most significant sleep and memory problems during the peri-menopausal period, changes in memory and sleep have also been observed across the menstrual cycle and during pregnancy. Here, we review current knowledge on the interrelationships among female sex hormones, sleep, and memory across the female lifespan, propose possible mediating and moderating mechanisms linking these variables and describe implications for ADRD risk in later life.

## Introduction

Globally, the number of individuals aged 65 and older is growing at an unprecedented pace—from 506 million in 2008 to an estimated 1.4 billion by 2040 (Cauley, 2012). As a result, aging-related changes in health, including cognitive decline and dementia are rising public health challenges. The worldwide prevalence of dementia in people over the age of 60 was estimated to be 5%–7% in 2010 and is expected to double every 20 years, with 115.4 million individuals projected to have dementia by 2050 (Prince et al., 2013). Alzheimer’s disease (AD) is the most common cause of dementia, accounting for up to 70% of cases (World Health Organization, 2021). The prevalence of sleep disturbance also increases with age, with 36%–69% of older adults reporting sleep complaints (Foley et al., 2004). An extensive literature documents the key role of sleep in memory consolidation in healthy individuals (Stickgold, 2005; Rasch and Born, 2013), and recently, an important link has been established between sleep and cognitive impairment due to AD and related dementias (ADRD) (Merlino et al., 2010; Peter-Derex et al., 2015). Greater sleep fragmentation, excessively short and long sleep duration, and sleep disorders have all been linked to an increased risk of ADRD (Ju et al., 2014; Peter-Derex et al., 2015). Indeed, sleep deprivation increases the interstitial fluid and cerebrospinal fluid levels of amyloid beta (Aβ) and tau, as well as brain Aβ deposition—a key pathological finding in AD (Kang et al., 2009; Holth et al., 2019). Further, preclinical studies suggest that Aβ accumulation may have a direct negative impact on sleep (Ju et al., 2014).

Although advanced age is one of the most potent risk factors for memory deficits, sleep disturbances, and AD, biological sex also plays an important role (World Health Statistics, 2017). Women have an increased risk of ADRD compared to age-matched men, as well as faster cognitive decline after diagnosis (Li and Singh, 2014; Mazure and Swendsen, 2016). Further, meta-analyses indicate that, compared to men, women have up to double the risk of sleep disturbances and insufficient sleep across the lifespan (Soares, 2005; Zhang and Wing, 2006).

Other excellent reviews have surveyed the literature concerning the interrelationships of sex hormones, sleep, and cognition (Gervais et al., 2017; Baker et al., 2019; Hajali et al., 2019; Brown and Gervais, 2020). In this review, we outline sex differences in memory and sleep, and expand on prior work describing changes in sleep and memory that occur during periods of hormonal changes, including the menstrual cycle and menopause. Further, we discuss sleep, memory, and their relationship during pregnancy, a period characterized by extremely high levels of female sex hormones. Finally, we discuss the interrelationships among sleep, memory, and hormones, and their implications for ADRD. Please note that we use the term “sex” to refer to the “assigned” sex of female or male at birth, though we acknowledge the complexity of factors such as chromosomes, genitalia, and hormones in this determination (Elsesser, 2020).

## Sex Differences in Sleep, Memory, and Neural Plasticity

### Sleep

Sleep is divided into two main categories: non-rapid eye movement (NREM) sleep and REM sleep. NREM sleep consists of stages N1, N2, and N3. N3 is also known as slow-wave sleep (SWS) and was divided into stages 3 and 4 in earlier frameworks (Rechtschaffen and Kales, 1968; Iber et al., 2007; Moser et al., 2009). Each stage is characterized by distinct neurophysiological features (Table 1). We cycle through these sleep stages repeatedly across the night, with the duration of time spent in each stage changing across cycles. In particular, the proportion of time spent in N3, the deepest stage of sleep, decreases over the course of the night, while the proportion of time spent in REM sleep, in which dreams typically occur, increases over the course of the night. *Sleep architecture* refers to this organization of sleep stages across the night (Paul et al., 2006; Chokroverty, 2017).

**Table 1:** Sleep stages and their electroencephalographic features.

**Category**	**Stages**	**EEG**	**Description**
**Non rapid eye movement (NREM) sleep**	**Stage 1 (N1)**	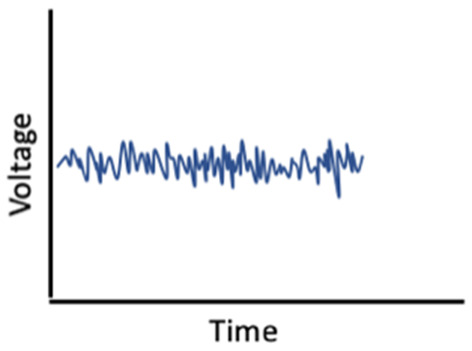	Lightest sleep stage, a transition between wake and sleep Easy to rouse a sleeper in N1 Wave Type: Alpha (8–12 Hz) Theta (47#x02013;7 Hz)
	**Stage 2 (N2)**	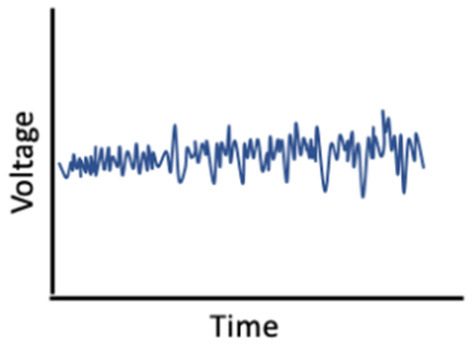	Heart rate and body temperature drop Features sleep spindles and K-complexes (see below) Harder to rouse than in N1 Wave type: Theta (4–7 Hz) Spindles (117#x02013;15 Hz); K-Complexes (12–15 Hz)
	**Stage 3 (N3)**	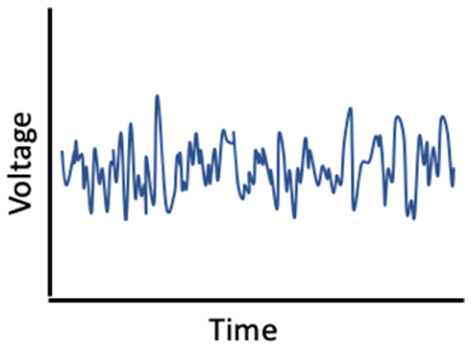	Also known as slow-wave sleep (SWS) Deepest sleep stage, hardest to rouse sleepers in N3 Wave type: Delta (0.5–2 Hz) Theta (4–7 Hz) Amplitude: >75μV
**Rapid eye movement (REM) sleep**	**REM**	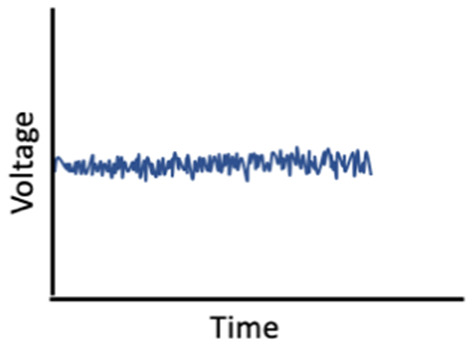	Rapid eye movement Most muscles paralyzed More desynchronized neuronal activity Wave type: Theta (4–7 Hz) Beta (15–35 Hz)
**Sleep electroencephalogram (EEG) features**	**Sleep spindles**	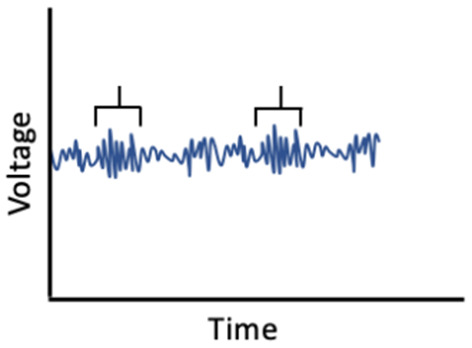	Brief spurts of synchronized brain activity in thalamocortical circuits Observed during N2 sleep Frequency: (11–15 Hz)
	**K complexes**	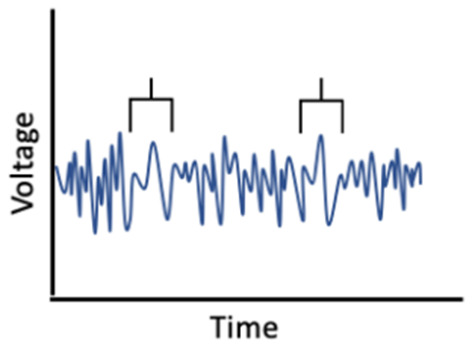	Waveforms primarily observed in the cortex during N2 sleep Frequency: (12–15 Hz)
	**Slow oscillations**	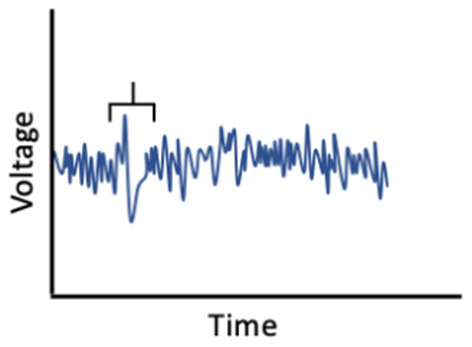	Reflect alternations between hyperpolarization (cortical neurons inactive) and depolarization (cortical neurons highly active) Observed during N3 sleep Frequency: (0.5–1 Hz)

*Note: Descriptions, frequencies (Hz), and amplitudes (mV) from Carskadon and Dement (2011); Berry et al. (2015); and Nayak and Anilkumar (2022)*.

Sex differences in sleep are evident across the lifespan, and other published reviews have covered this topic in detail (Carrier et al., 2017; Dib et al., 2021). In brief, compared to men, women report a greater number of sleep disturbances (e.g., lower subjective sleep quality and more frequent arousals) and have a higher prevalence of insomnia disorder (Mong and Cusmano, 2016). Effects of estradiol and progesterone on the structure and function of brain regions important for controlling sleep and wake may be partially responsible for these differences (Deurveilher et al., 2009).

The ventrolateral preoptic nucleus (VLPO) is a group of neurons located in the anterior hypothalamus that inhibit major arousal systems during sleep through the release of wake-inhibiting neurotransmitters and peptides [e.g., gamma-aminobutyric acid (GABA) and galanin] to histaminergic neurons (Saper et al., 2005). The VLPO is thought to be stimulated by the somnogens prostaglandin D2 (PGDS) and adenosine, which accumulate during wake (Scammell et al., 1998; Nagata and Urade, 2012). In female rodents, estradiol leads to inhibition of PGDS in the VLPO, which inhibits sleep and increases levels of physical activity during wake (Mong et al., 2003a, b; Hadjimarkou et al., 2008). Further, estradiol may inhibit adenosine receptors in the VLPO and preoptic area (POA) to decrease sleepiness and increase physical activity during wake (Ribeiro et al., 2009). One study found that ovariectomized female rats receiving estradiol with and without crystalline progesterone had less REM and NREM sleep, and those receiving only the crystalline progesterone treatment had less REM sleep compared to gonadectomized rats receiving no hormonal treatment (Deurveilher et al., 2009). Another neural structure implicated in sleep-wake regulation is the parafacial nucleus of the medulla, which projects to the medial parabrachial nucleus and releases GABA to inhibit wakefulness (Anaclet et al., 2012). The parabrachial nucleus, which plays a regulatory role in respiration, contains numerous estrogen receptors, indicating the potential influence of estrogen on parabrachial nuclear functioning (Shughrue et al., 1997; Saleh and Connell, 2003; Chamberlin, 2004). Studies have shown that the inhibitory effect of estrogens on the parabrachial nucleus could be mediated by GABA receptors and may, therefore, have negative implications for sleep and lead to a higher risk of sleep-disordered breathing (SDB) during times of hormone fluctuation, especially high estradiol concentrations (e.g., pregnancy) (Saleh and Saleh, 2001; Saleh and Connell, 2003). In contrast, clinical studies suggest that estradiol and progesterone promote sleep in women, as discussed below. However, there are no investigations on the direct effects of estradiol and progesterone on the VLPO and other sleep/wake structures in humans.

Sex hormones may also affect sleep indirectly, by altering *circadian rhythms* (Paul et al., 2006)—physiological processes that follow an approximately 24-h cycle (e.g., changes in core body temperature, alternation of sleep, and wake), and continue under constant conditions (e.g., in dim light), and are synchronized to the light-dark cycle of the environment. In mammals, the suprachiasmatic nucleus (SCN) in the hypothalamus is the central “pacemaker” governing peripheral body clocks (Hastings et al., 2018). There are prominent sex differences in factors that may affect circadian rhythms. For example, females have a larger SCN with a greater number of estrogen and progesterone receptors compared to males, and males have a greater number of androgen receptors in the SCN compared to females (Curran-Rauhut and Petersen, 2002; Kruijver and Swaab, 2002; Karatsoreos et al., 2007; Vida et al., 2008; Bailey and Silver, 2014). These factors suggest a direct pathway through which these sex hormones may modulate circadian rhythms. Although intriguing links may exist among sex hormones, circadian rhythms, and memory, a more detailed discussion of circadian rhythms is beyond the scope of the present review.

### Memory and Neural Plasticity

It is generally accepted that there are three types of memory: sensory memory, short-term memory, and long-term memory (Camina and Güell, 2017). In this review, we primarily consider long-term memory, given it is strongly affected by sleep and is the cognitive domain most studied in the context of AD. Long-term memory can be categorized into declarative memory, which consists of episodic and semantic memories and can consciously be accessed, and non-declarative memory, which is thought to be used outside of conscious awareness and includes procedural memory, conditioning/associative learning, non-associative learning, and priming (Stickgold, 2005; Camina and Güell, 2017). Episodic memories contain information about personal experiences, whereas semantic memory relates to factual information that can be learned (Dickerson and Eichenbaum, 2010). Procedural memory is the ability to recall motor tasks and skills, conditioning is the learned association of two items, non-associative memory refers to the decrease in a response (habituation) or increase in a response (sensitization) after repeated exposure to a stimulus, and priming refers to the effect of exposure to one stimulus or the response to other stimuli presented at a later time (Camina and Güell, 2017). These types of long-term memories are encoded and consolidated using distinct hippocampal circuits and brain structures. Briefly, declarative memories utilize areas of the temporal lobe and diencephalon while nondeclarative memories utilize the striatum and cerebellum (procedural), cerebellum and amygdala (conditioning), reflex pathways (non-associative), and the neocortex (priming) (Squire and Zola, 1996; De Zeeuw and Ten Brinke, 2015). For consistency and conciseness, this review focuses on the role of the hippocampus and prefrontal cortex as they relate to memory, although we acknowledge that other brain regions also play important roles. There are also broader types of memory, including *emotional memory*, which involves learned behavior as a result of an emotional experience; *verbal memory*, which is learned information related to words or language presented in a visual or auditory context; and *spatial memory*, which is knowledge related to spatial location (Tatsumi and Watanabe, 2009; Dickerson and Eichenbaum, 2010; Baghdadi et al., 2021).

Sex differences have been identified in various memory domains and have been described extensively in other reviews (Hamson et al., 2016; Hampson, 2018, 2020). For example, on average, women outperform men on tests of episodic memory, verbal memory, and emotional memory (Herlitz and Rehnman, 2008; Andreano and Cahill, 2009), whereas men fare better on tests of spatial recall (Weiss et al., 2003; Shah et al., 2013). It is important to note, however, that some studies have found no significant sex differences in episodic and semantic memory domains (Banta Lavenex and Lavenex, 2010; Cheke and Clayton, 2013; Nori et al., 2018); thus, the extent of these differences remains debated.

Memory consolidation is the process by which short-term memories stored in the hippocampus become long-term memories, through “neural replay” between the hippocampus and the neocortex (Squire et al., 2015). This process is greatly facilitated by sleep (Stickgold, 2005). Sleep-dependent memory consolidation relies on discrete electrophysiological features of sleep (Diekelmann and Born, 2010b). SWS plays a central role in memory consolidation (Born, 2010), and memories are subsequently fortified during REM sleep (Diekelmann and Born, 2010a). In addition, sleep spindles—bursts of neural oscillations across thalamic circuits—are thought to promote synaptic plasticity, and memory reinforcement, possibly through calcium-mediated signaling cascades (Cox et al., 2012; Genzel et al., 2012; Aeschbach and Santhi, 2013; Rasch and Born, 2013; Squire et al., 2015; Baker et al., 2019; Fernandez and Lüthi, 2020). Although sex differences in sleep are described in greater detail below, it is important to note that adult women exhibit greater spindle frequency and SWS compared to men (Fukuda et al., 1999; Huupponen et al., 2002; Ujma et al., 2014). These differences may account for findings of better sleep-dependent memory consolidation in women, compared to men, in multiple memory domains (e.g., verbal, emotional); however, results concerning these sex differences are inconsistent (Backhaus and Junghanns, 2006; Genzel et al., 2012). Sex differences are also present in the cellular mechanisms underlying both sleep-dependent memory consolidation and non-sleep-specific consolidation (McDevitt et al., 2014). Compared to males, female rats and humans demonstrate greater synaptic plasticity in the hippocampus and neocortex (Juraska, 1991; Hyer et al., 2018). Moreover, exogenous estradiol and progesterone exposure in the hippocampus may induce increased neurogenesis and spine density in the female rat brain (Woolley and McEwen, 1993; Barker and Galea, 2008). Given that estradiol and progesterone may enhance memory retention by altering synaptic plasticity in brain regions critical to memory processes (i.e., hippocampus and prefrontal cortex) (Foy et al., 2010; Frankfurt and Luine, 2015), sex differences may be driven by female sex hormones (Morse et al., 1986; Sandstrom and Williams, 2001). As we discuss in further detail below, fluctuations in estrogens and progesterone levels during the menstrual cycle, pregnancy, and menopause may result in changes in memory performance across the adult female lifespan and may account, in part, for the observed sex differences in ADRD prevalence in later life (Peterson and Tom, 2021).

Synaptic plasticity and neurogenesis in the hippocampus and prefrontal cortex play a crucial role in cognitive processes, especially memory (Frankfurt and Luine, 2015). Studies have found that estradiol exposure can lead to decreased cell death and increased spine density in hippocampal cornuammonis (CA1) pyramidal neurons and in the prefrontal cortex (Woolley and McEwen, 1993; Pozzo-Miller et al., 1999; Hao et al., 2006; Barker and Galea, 2008; Khan et al., 2013). For example, mice genetically modified to be completely estrogen-deficient showed increased cell death in the prefrontal cortex, which was reduced following 17β-estradiol administration (Hill et al., 2009). These findings suggest that estradiol can reduce cell death and promote the formation of new synapses, which is essential for new learning and reinforcement of memory. Estradiol is also thought to impact synaptogenesis through N-Methyl-D-aspartate (NDMA) receptors, cholinergic pathways, and dopaminergic pathways (Brett and Baxendale, 2001; Romeo et al., 2005; Almey et al., 2015; McEwen and Milner, 2017). On the other hand, administration of progesterone decreases spine density in the hippocampus and may attenuate estradiol’s synaptogenic effect. In addition, administration of a progesterone antagonist may result in persistently greater spine density following exposure to estradiol (Woolley and McEwen, 1993). Therefore, progesterone could help minimize unnecessary synaptic connections and be involved in synaptic stabilization to maintain the efficiency of the brain for memory function.

Long-term potentiation (LTP) is the strengthening of the excitatory post synaptic response as a result of repeated stimulation and is believed to be a key process underlying synaptic plasticity and memory (Lynch, 2004). Estradiol is thought to promote LTP by reducing the threshold for inducing a response as well as increasing the amplitude of stimulation (Kramár et al., 2013). Estrogens are also thought to activate receptors for brain-derived neurotrophic factor (BDNF), which has been found to positively correlate with LTP stability (Rex et al., 2006; Kramár et al., 2013). Further, estradiol and pregnenolone sulfate, a precursor to steroid hormones, may increase LTP by modulating estrogen receptor α in females and NMDA receptors in both sexes to produce an excitatory response in the hippocampus (Foy et al., 1999; Sliwinski et al., 2004; Wang et al., 2018). Additionally, there seems to be a dose-dependent effect, as Foy et al. (1999) observed seizure effects of estradiol at doses above 10 nM, whereas lower doses increased the amplitude of the excitatory postsynaptic potentials mediated by NMDA receptors without inducing seizures. Progesterone, on the other hand, is thought to have an inhibitory effect on LTP. Foy et al. (2008) found that administration of progesterone to rat hippocampal slices decreased the number of excitatory post-synaptic potentials, and thus negatively affected LTP. The authors speculated that this effect maybe due to the activation of inhibitory GABA receptors (Foy et al., 2010).

Sleep is also thought to augment LTP. Sleep deprivation is thought to reduce the excitability threshold of synapses (Abel et al., 2013). Prince et al. (2014) found that sleep deprivation of 3 h initiated 1–4 h following a spatial task resulted in decreased LTP in the CA1 area of the hippocampus in rodents, yet this effect was not observed if sleep deprivation began immediately following the task. This indicates the possibility of a critical window during which sleep is important to memory consolidation. Notably, Romcy-Pereira and Pavlides (2004) found that short-term REM sleep deprivation decreased LTP in the hippocampus but increased LTP in the medial prefrontal cortex. The researchers hypothesized that long-term depression (e.g., the reduction of synaptic strength), not LTP, may be important for memory function in the prefrontal cortex (Romcy-Pereira and Pavlides, 2004). In relation to NREM sleep, Rosanova and Ulrich (2005) demonstrated that the neuronal firing pattern of sleep spindles can evoke LTP in neocortical pyramidal cells. Furthermore, Wei et al. (2018) demonstrated that the firing pattern of spindles in NREM sleep increased synaptic weights in cortical neurons that positively correlated with memory performance after sleep, suggesting that sleep spindles are important for memory consolidation.

## Interrelationships Among Female Sex Hormones, Sleep, and Memory in Women

Given the potential role of sex and sex hormones on memory performance, sleep, and synaptic plasticity, we next review current knowledge of the interrelationships of female sex hormones, sleep, and memory across the adult female lifespan.

### The Menstrual Cycle

The average menstrual cycle lasts 28 days and features characteristic changes in female sex hormones, regulated by feedback mechanisms within the hypothalamic-pituitary-ovarian axis (Reed and Carr, 2018). The menstrual cycle is divided into two phases: (1) the follicular phase, which begins with the onset of menses; and (2) the luteal phase, which starts with ovulation. At the beginning of the follicular phase, the anterior pituitary gland releases follicle-stimulating hormone and luteinizing hormone, which stimulate the ovaries to develop follicles and produce estrogen. There are three main types of endogenous estrogens produced by the ovaries: estrone (E1), estradiol (E2, or estradiol-17β), and estriol (E3). Estradiol (E2) is the most potent estrogen in its effects on estrogen receptors throughout the brain and body and is the most prevalent estrogen during reproductive years. Conversely, E3 is the least potent estrogen and is most prevalent during pregnancy (Bulun, 2016). During menopause, E1 becomes the predominant estrogen due to the reduction in ovarian production of E2. The early follicular phase is the least hormonally active period, with low estradiol and progesterone levels. Estradiol levels rise rapidly and peak in the late follicular phase. The luteinizing hormone surge follows 1 day after the estradiol peak and results in ovulation. The luteal phase is marked by a rise in progesterone levels and a second rise in estradiol levels during the mid-luteal phase. By the end of the luteal phase, if no fertilization has occurred, there is a progressive decline in progesterone and estradiol levels, resulting in the onset of menses (Reed and Carr, 2018).

#### The Menstrual Cycle and Memory

Performance on memory-related tasks differs based on the menstrual phase. For example, women perform better on tasks assessing procedural memory, verbal memory, nondeclarative memory, and emotional memory around the time of ovulation, when estradiol levels are elevated (Hampson, 1990a; Maki et al., 2002; Sundström Poromaa and Gingnell, 2014). Further, Ertman et al. (2011) found that levels of progesterone during the menstrual cycle positively correlate with performance on emotional memory free recall and recognition tests. Performance on spatial memory tasks, however, decreases during periods of heightened estradiol and progesterone levels (Hampson, 1990a, b; Phillips and Silverman, 1997; Hausmann et al., 2000). Together, these findings suggest that gonadal hormones contribute to sex differences in memory performance, and that distinct memory domains respond differently to altered hormone levels.

Estrogens and progesterone also influence hippocampal and prefrontal cortex functions that subserve memory. A functional magnetic resonance imaging (fMRI) study found that, when performing a verbal memory task, higher endogenous estradiol concentrations seen in the late follicular phase correlated with greater activation in the left prefrontal cortex (Craig et al., 2008). Konrad et al. (2008) also found greater prefrontal activation while performing a semantic retrieval task during the mid-luteal phase compared to the early follicular phase, which was highly correlated with endogenous estradiol and progesterone levels. Further, cortical activation patterns in the hippocampus and prefrontal cortex associated with verbal and spatial memory are larger in women during the late follicular phase, when estradiol levels are high, compared to women in the earlier follicular phase and with men (Dietrich et al., 2001). While other studies have demonstrated decreased spatial memory performance during menstrual phases with high estradiol levels (Hampson, 1990a, b; Phillips and Silverman, 1997; Hausmann et al., 2000), the differences in results may be related to different studies testing different aspects of spatial memory with different cortical mechanisms (Linn and Petersen, 1985; Postma et al., 1999). Thus, further studies testing different types of memory are needed to better determine which aspects of memory are influenced by these hormones.

The notion that hormonal fluctuations may alter brain function is further supported by evidence of neuronal and structural brain changes in specific phases of the menstrual cycle in humans and in the estrous cycle of the rat. Notably, rodents do not have a menstrual cycle, but rather an estrous cycle that lasts 4–6 days and consists of four phases: proestrus (follicular phase equivalent), estrus (ovulation phase equivalent), metestrus (early luteal phase equivalent), and diestrus (late luteal/menses phase equivalent) with similar fluctuations in hormones as in the human menstrual cycle (Ajayi and Akhigbe, 2020). In rats, dendritic spine density in the hippocampus and overall gray matter volume have been shown to peak around the time of ovulation, paralleling a peak in estradiol levels (Woolley et al., 1990; Woolley and McEwen, 1993; Yankova et al., 2001) and in humans (Hagemann et al., 2011). In women, Protopopescu et al. (2008) found that hippocampal gray matter volume was higher during the late follicular phase compared to those in the late luteal phase. In another study, Lisofsky et al. (2015) observed higher hippocampal gray matter volume and increased functional connectivity between the hippocampus and parietal lobe during the late follicular phase, compared to the early follicular phase. Also, using magnetic resonance imaging, Barth et al. (2016) observed microstructural changes in the hippocampus across the menstrual cycle. With diffusion tensor imaging methods, the investigators found a positive association between serum estradiol concentration and fractional anisotropy and a negative association between estradiol and radial diffusivity (Barth et al., 2016). Together these findings suggest that estradiol may enhance hippocampal gray matter volume and reinforce white matter integrity.

Evidence suggests that hormone levels must be within a specific range to facilitate neurological changes. For example, intermediate concentrations of exogenous estradiol increased hippocampal cell proliferation important for episodic memory in ovariectomized rats, whereas excessively low or high levels did not (Tanapat et al., 2005). Further, administering exogenous progesterone alongside exogenous estradiol to ovariectomized rats attenuated the neuronal propagation observed when administering estradiol alone (Tanapat et al., 2005). Additionally, after applying a progesterone receptor antagonist, the reduction in spine density seen with exogenous progesterone was eliminated (Woolley and McEwen, 1993). Moreover, BDNF, which mediates neuronal plasticity, peaks alongside estradiol during the late follicular and mid-luteal phases (Begliuomini et al., 2007). Therefore, estradiol may promote BDNF-mediated neural plasticity and enhance performance in various memory domains, whereas progesterone may modulate estradiol’s effects and prevent neuronal overgrowth.

These studies illustrate the influence of endogenous and exogenous forms of estrogens and progesterone on brain structure and function that may underlie the memory performance differences seen across the menstrual cycle. Although the fluctuating concentrations of estradiol and progesterone during the reproductive years may not produce neurological changes that significantly impair performance, more extreme fluctuations (e.g., in pregnancy and menopause) may have a more prominent effect on memory consolidation and performance, as discussed below. Importantly, hormonal fluctuations may also indirectly affect memory across the menstrual cycle by altering sleep quality and quantity—a topic to which we now turn.

#### The Menstrual Cycle and Sleep

Sleep duration and quality fluctuate across the menstrual cycle. Women report lower sleep quality during the early follicular and late luteal phases, which correspond to the nadir of estradiol and progesterone levels (Baker and Driver, 2004). Indeed, in women, a reduction in actigraphy-measured sleep efficiency and total sleep time has been observed in the late luteal phase prior to menstruation, when progesterone levels drop (Zheng et al., 2015). In addition, women using oral or vaginally administered contraceptives containing progestin only, or a combined treatment with estradiol, all reported better subjective sleep quality (Guida et al., 2020). Baker et al. (2001) found that women taking various combined oral contraceptives of estradiol and progestin had greater stage 2 sleep compared to placebo or naturally-cycling controls, yet naturally-cycling women in the luteal phase spent more time in SWS compared to those taking oral contraceptives. Therefore, endogenous and exogenous estradiol and progesterone may have different effects on sleep (Baker et al., 2001). Progesterone may exert its effects through GABA receptors located in sleep-wake structures such as the VLPO and POA of the hypothalamus. Progesterone metabolites such as 5α-dihydroprogesterone and 3α-hydroxy-dihydroprogesterone may also enhance sleep by stimulating GABA receptors (Majewska et al., 1986; Gottesmann, 2002; Wang, 2011). Exogenous progesterone decreased wakefulness and latency to NREM sleep and was associated with GABA agonistic metabolites in rats in a dose-dependent manner; however, 5α and 5β pregnanolone did not alter NREM sleep (Lancel et al., 1996). Taken together, both estradiol and progesterone appear to promote sleep quality, with both hormones increasing stage 2 sleep and progesterone increasing SWS. Therefore, progesterone and synthetic progestins may differentially affect sleep (Pluchino et al., 2006). This is important to consider when interpreting the effects of endogenous and exogenous forms of progesterone.

Sleep spindles are one of the hallmark EEG features of stage 2 sleep, and alterations in spindles are among the most-studied aspects of sleep micro-architecture across the menstrual cycle (Genzel et al., 2012). Estradiol and progesterone may both promote sleep spindles, as the highest spindle density is seen during the luteal phase when estradiol and progesterone levels are high. Conversely, the lowest sleep spindle density occurs following menses, when these hormones are low (Ishizuka et al., 1994; Driver et al., 1996; Genzel et al., 2012; Plamberger et al., 2021). In addition, women spend the most time in stage 2 sleep during the early luteal phase (Driver et al., 1996). Taken together, these studies suggest a positive relationship between higher estradiol and progesterone levels seen in the luteal phase and the amount of stage 2 sleep.

Differences in SDB also have been observed across the menstrual cycle, with women exhibiting greater upper airway resistance and a greater number of respiratory events (e.g., hypopneas, apneas, related arousals) during the follicular phase, when progesterone levels are low than in the luteal phase, when levels are high (Driver et al., 2005). Additionally, a study of women undergoing evaluation for daytime sleepiness and suspected SDB found that those with obstructive sleep apnea (apnea-hypopnea index greater >10) had lower progesterone and estradiol levels than those without, after accounting for age, menstrual phase, and menopausal status (Netzer et al., 2003), suggesting that progesterone may protect against SDB. Findings, however, are inconsistent (Stahl et al., 1985), perhaps due to inter-individual differences in gonadal hormone levels across the menstrual cycle (Baker and Lee, 2018). Hormonal fluctuations across a single menstrual cycle are insufficient to produce clinically significant SDB or other sleep disorders, yet it is unknown whether repeated exposure to these variations accumulates across the reproductive years to exert effects on later sleep and brain health.

Overall, findings suggest that fluctuations of estradiol and progesterone alter sleep, with better sleep quantity and quality generally seen during the luteal phase, characterized by higher concentrations of estradiol and progesterone.

#### Links of Sleep and Memory Across the Menstrual Cycle

A handful of studies have compared sleep-dependent memory consolidation between men and women, including across the menstrual cycles of female participants. Backhaus and Junghanns (2006) found that women had better post-nap declarative memory performance compared to men, whereas Genzel et al. (2012) found that men had better post-nap declarative and motor memory performance compared to women. This conflicting finding may be due, in part, to differences in the menstrual phase of the women selected for each study. Whereas Genzel et al. (2012) only included women during menses, when estradiol and progesterone levels are low, Backhaus and Junghanns (2006) did not control for the menstrual phase. Therefore, better sleep during the luteal phase for some women in the Backhaus and Junghanns study may have partially accounted for the results. Moreover, Genzel et al. (2012) observed that after learning motor and declarative memory tasks, men had an increase in spindle density during a nap, whereas women only exhibited this increase if they were in the mid-luteal phase. They also found that women in the luteal phase and men had a better post-nap performance on these tasks than women in the follicular phase. These findings suggest that hormonal changes associated with the menstrual cycle alter sleep spindle density and thereby affect sleep-dependent memory consolidation. The authors also found that, during the luteal phase, higher estradiol levels were associated with better sleep-dependent declarative memory, whereas higher progesterone levels were associated with greater motor memory consolidation during sleep (Genzel et al., 2012), suggesting these hormones affect performance in different memory domains. Sattari et al. (2017) similarly found a menstrual phase-dependent effect of a nap on memory consolidation of face-name associations. Overall, women had lower performance on a recognition memory task post-nap during their perimenses phase (5 days before to 6 days after the start of menses) compared to their post-nap performance outside of the perimenses phase and that of men. Among the women in the perimenses phase, sleep spindles during the nap were associated with higher memory performance, whereas slow oscillations were not. On the other hand, among women in the non-perimenses phase, slow oscillations rather than sleep spindles during a nap were associated with higher memory performance. Both sleep spindles and slow oscillations are important for memory consolidation and may have specific effects at different points of the menstrual cycle based on estradiol and progesterone concentrations. Further, while sleep spindles may promote memory consolidation regardless of the menstrual phase, spindles and thus memory may be enhanced directly by estradiol and progesterone fluctuations, or indirectly through increased slow oscillation coupling during the luteal phase.

In addition, Plamberger et al. (2021) compared sleep and memory performance among women taking a combined estradiol and progestin pill (dienogest and ethinylestradiol or levonorgestrel and ethinylestradiol), naturally cycling women in their luteal phase, and naturally cycling women in their follicular phase. They found that declarative memory recall improved post-sleep in all women, but overnight improvement was greater in naturally cycling women in their luteal phase and in women taking the combined estradiol/progestin pill, compared to naturally cycling women in their follicular phase (Plamberger et al., 2021). Further, greater sleep spindle density was seen in women taking the combined estradiol/progestin pill and in naturally cycling women during the luteal phase compared to naturally cycling women in the follicular phase (Plamberger et al., 2021). Given that combined estradiol/progestin pills contain high levels of synthetic estradiol and progestin and the luteal phase is characterized by high endogenous levels of these hormones, this further implicates estradiol and progesterone in memory consolidation.

In sum, the effects of sleep on memory formation and consolidation vary depending on the menstrual phase, and both endogenous and exogenous gonadal hormone levels may be driving these differences. During the menstrual cycle, increases in estradiol and progesterone appear to enhance sleep quantity and quality; however, beneficial effects may only result from mild to moderate increases in hormone levels, and markedly elevated hormone levels during pregnancy may have detrimental effects on memory and sleep, as discussed in the next section.

### Pregnancy

During pregnancy, steadily increasing levels of gonadal hormones, including estrogens and progesterone, maintain pregnancy, support fetal development, and prepare the mother’s body for childbirth (Mesiano, 2001). During pregnancy, the major estrogen in maternal circulation is estriol (E3), which is produced by the placenta *via* the conversion of 16-hydroxy-dehydroepiandrosterone sulfate (DHEA-S) to androgens and then aromatized to estriol (Liu et al., 2013). Further, progesterone is needed for proper implantation of the embryo and acts to inhibit the mother’s immune response to fetal antigens (Kumar and Magon, 2012). Both estrogen and progesterone are initially produced by the ovaries and corpus luteum during the first trimester, and then primarily produced by the placenta for the rest of pregnancy (Mesiano, 2001). By the end of the third trimester, both estrogen and progesterone levels are exceptionally high compared to levels at the beginning of pregnancy and during the menstrual cycle (Tulchinsky et al., 1972).

#### Pregnancy and Memory

Over 50% of women report memory problems or other cognitive deficits during pregnancy (Janes et al., 1999; Davies et al., 2018). This has led to the colloquial phrase “pregnancy brain” or “baby brain” (Brown and Schaffir, 2019). There is still debate, however, over whether there is a difference in memory performance during pregnancy; some have suggested that cognitive deficits may reflect a cultural stereotype, rather than true deficits (Crawley et al., 2008). Studies have resulted in mixed findings, with some suggesting cognitive impairments and others finding no change in memory performance during pregnancy. For example, Logan et al. (2014) found that while pregnant women had more subjective memory deficits during the third trimester and 3 months postpartum, they did not differ significantly on objective memory tests. Another study found that only nondeclarative memory, tested with an objective word completion priming task, was lower in pregnancy and correlated with subjective decreases in memory performance (Brindle et al., 1991).

Studies in rodents, however, have shown that early pregnancy is associated with memory enhancement, and that later pregnancy is associated with impaired memory performance (Galea et al., 2000; Wilson et al., 2011a; Workman et al., 2012). Because endogenous estrogens and progesterone levels are relatively low in early pregnancy compared to later pregnancy, these findings suggest that moderately elevated levels of progesterone and estrogens are beneficial for memory, whereas markedly high levels are detrimental. Indeed, studies found that pregnant women in their third trimester had lower verbal memory performance compared to pregnant women in their first trimester and non-pregnant controls; moreover, lower performance was associated with higher endogenous estradiol and progesterone levels seen in the third trimester (Glynn, 2010; Wilson et al., 2011a). Despite higher levels of estradiol being associated with better performance during the menstrual cycle, these studies suggest that estradiol improves these domains up to a threshold that is exceeded during pregnancy.

Pregnancy-related changes in cognition and memory are paralleled by changes in brain structure and function. Animal and human studies suggest pregnancy-related changes occur in hippocampal and prefrontal cortex structure and function (Henry and Rendell, 2007; Hoekzema et al., 2017; Barba-Müller et al., 2019). Greater spine density in the CA1 area of the hippocampus and in the prefrontal cortex has been observed in pregnant compared to nonpregnant rats (Kinsley et al., 2006; Cabrera-Pedraza et al., 2017); however, other studies have found no significant pregnancy-related differences in hippocampal brain volumes and even lower amounts of hippocampal neurogenesis among pregnant rats (Galea et al., 2000; Rolls et al., 2008). On the other hand, a neuroimaging study that followed rodents from 3 days before (baseline) to 17 days after mating (equivalent to human third trimester) found structural changes in the hippocampus (Chan et al., 2015). Additionally, in rodents, monoamine neurotransmitter activity decreases in the prefrontal cortex but increases in the hippocampus during pregnancy (Macbeth et al., 2008). This may help explain the more pronounced deficits in domains of memory subserved by the cortex (e.g., episodic memory) in comparison to other forms of memory (e.g., procedural, visual memory) (Wilson et al., 2011a). Overall, results regarding the hippocampal brain changes during pregnancy in rodents remain inconclusive; some have found changes that would indicate pregnancy-induced improvements in memory, while others the opposite. Nonetheless, findings suggest memory performance may differ by pregnancy stage, with the lowest performance seen in the third trimester as sex hormone levels peak.

Although only a few human studies have investigated structural and functional brain changes during pregnancy, they generally suggest a reduction in volumes in several regions during pregnancy which, depending on the brain region, may continue into the postpartum period or return to similar baseline levels. Hoekzema et al. (2017) found reductions in prefrontal gray matter volume, left hippocampal volume, cortical thickness, and surface area (which is believed to be highly sensitive to female sex hormones) shortly after birth compared to pre-pregnancy while nulliparous women demonstrated no changes. Conversely, volumes in areas associated with maternal behavior such as the hypothalamus, amygdala, and substantia nigra have increased shortly after birth (Kim et al., 2010; Barba-Müller et al., 2019). Studies have also shown a decrease in total brain volume during pregnancy that typically reverses in postpartum (Oatridge et al., 2002). However, other studies have found these changes may persist and extend into the postpartum period. Kim et al. (2018) found that prefrontal cortex cortical thickness was positively correlated with months since birth in the first 6 months postpartum. These results may be related to stabilizing hormone levels, which are believed to return to baseline levels around 6 months postpartum. Although, the subregions of the hippocampal gray matter volume return to pre-pregnancy levels, reductions in prefrontal gray matter are maintained up to 2 years postpartum in humans (Hoekzema et al., 2017; Luders et al., 2021). These persistent structural changes may cause residual effects on memory performance 3 months post pregnancy (Glynn, 2010). Further, another longitudinal study rescanned a subsample from the Hoekzema et al. (2017) study, which found reductions in prefrontal gray matter volume, cortical thickness, and surface area 2 years postpartum, again at 6 years postpartum, and found many of the observed gray matter reductions seen shortly after birth were still present 6 years later (Martínez-García et al., 2021). The researchers also commented that while Oatridge et al. (2002) observed an increase in brain volume 6 months postpartum, they did not include a control group of non-pregnant women or consider the number of previous pregnancies to better account for the impact of age and hormonal sensitivity (Martínez-García et al., 2021), which may also be extended to the Kim et al. (2010) study. Taken together, these findings suggest that pregnancy could lead to longstanding and perhaps permanent changes to the maternal brain.

Other researchers have suggested a beneficial effect of pregnancy later in life. In rodents, pregnancy may cause sustained high levels of dendritic density and length in the prefrontal cortex, and increased neurogenesis in the hippocampus (Cabrera-Pedraza et al., 2017; Eid et al., 2019). Moreover, pregnancy-induced memory improvements continue into older age in rats (Love et al., 2005; Macbeth and Luine, 2010). Gatewood et al. (2005) found that rats that had either one or multiple pregnancies performed better on a spatial memory task compared to rats that had never been pregnant. Further, the researchers found that the multiparous rats exhibited less memory decline (and notably, less amyloid precursor protein buildup in the hippocampus) than both primiparous rats and nulliparous rats. This suggests that pregnancy, and perhaps the hormonal fluctuations associated with this time period, may have long-term protective effects on the brain. Evidence for alterations in female sex hormones as the mechanism is supported by similar results from studies in which rats received a pregnancy-mimicking regimen of estradiol and progesterone (Toffoletto et al., 2014).

Moving to human studies, in women, de Lange et al. (2020) used machine learning to identify brain regions susceptible to the effects of pregnancy on brain aging in a sample of almost 20,000 women. The authors found that a greater number of childbirths correlated with lower brain volume reductions, especially for limbic brain regions including the hippocampus. The researchers suggested that the pregnancy-related increases in female sex hormones, cortisol, and anti-inflammatory markers, which fluctuate dramatically during pregnancy and can alter neural plasticity, mediate the observed effects on brain aging (de Lange et al., 2020). On the other hand, while Ning et al. (2020) observed better visual and verbal performance and reduced brain aging in women who had been pregnant, they also found similar results in male partners, suggesting it is not pregnancy *per se*, but perhaps parenthood that drives these effects. Therefore, more studies are needed to understand the links between the biological aspects of pregnancy to changes in memory and brain aging. Lastly, one study found that pregnancy may be protective for the development of ADRD later in life. Fox et al. (2018) found that a greater number of cumulative months spent in the first trimester over the reproductive lifespan, including periods of miscarriages and terminations, was associated with lower ADRD risk; however, this association was not found for the third trimester, and the second trimester was not measured. Thus, elevated (as seen in the first trimester), but not extremely high estrogens and progesterone levels (as seen in the third trimester), may be responsible for the effects of pregnancy on lowered ADRD risk (Fox et al., 2018). Alternatively, the authors suggested the moderate protective effect of the first trimester may be due to pregnancy-induced alterations in immune function, specifically the increase in regulatory T cells during the first trimester. More studies are needed to investigate the possible role of immune response in early pregnancy in mitigating subsequent ADRD risk.

In sum, whereas findings concerning the effects of pregnancy on objective memory performance and brain structural and functional changes remain mixed in rodents and humans, a majority of studies of subjective memory performance suggest it decreases during pregnancy. In addition, brain changes during and after pregnancy may drive changes in the memory performance. Further longitudinal studies in rodents and humans are needed to characterize how pregnancy alters brain structure and function and how these changes affect memory during pregnancy, postpartum, and later in life. As we discuss in the section that follows, pregnancy is also associated with significant changes in another driver of memory: sleep.

#### Pregnancy and Sleep

Approximately 75% of pregnant women report poor sleep quality during pregnancy (Mindell et al., 2015). This can be due to factors resulting directly from pregnancy (e.g., frequent need to urinate, uncomfortable sleep positions) and to diagnosable sleep disorders (e.g., insomnia, sleep apnea, and restless legs syndrome) (Mindell et al., 2015). Facco et al. (2010) demonstrated that decreased sleep duration and sleep disturbances are more prevalent in the third trimester compared to the first. Along with greater physical discomfort, steadily increasing estrogens and progesterone concentrations may also degrade sleep in later pregnancy (Silvestri and Aricò, 2019). Changes in sleep architecture have also been observed across pregnancy. During the last 2 months of pregnancy, women may exhibit decreased REM sleep and greater wakefulness after sleep onset (Driver and Shapiro, 1992; Hertz et al., 1992). NREM sleep also decreases across pregnancy (Brunner et al., 1994; Lee et al., 2000). Further, Brunner et al. (1994) found reduced spectral power density in frequencies associated with sleep spindles during the third trimester compared to the first and second in the same women. One study found that greater wakefulness after sleep onset and less time spent in NREM sleep in the third trimester was associated with the increased progesterone levels typically seen during this stage of pregnancy (Wilson et al., 2011b). This is the opposite of what would be expected, given progesterone is thought to have sleep-inducing effects (Majewska et al., 1986), and suggests that extremely high progesterone concentrations could interfere with sleep. As stated by Wilson et al. (2011b), however, progesterone may not be the only factor responsible for these differences; other possible contributors include pregnancy-associated digestive issues, leg cramps, and backache.

Pregnancy can also affect respiration during sleep. Increased endogenous estrogen is thought to cause upper respiratory changes such as mucosal edema and rhinitis, which increase upper airway resistance and may promote SDB (Sharma and Franco, 2004; Morong et al., 2014). The risk of SDB also rises as pregnancy progresses, with the highest risk during the third trimester when estrogens peak (Facco et al., 2014; Pien et al., 2014). On the other hand, a recent study found that progesterone levels were lower in pregnant women with obstructive sleep apnea than those without (Lee et al., 2017), suggesting progesterone may protect against SDB. This is supported by animal studies identifying progesterone as a respiratory stimulant in rats (Bairam et al., 2015). Further, Brownell et al. (1986) found that SDB risk was lower during the third trimester, when progesterone levels are high, compared to postpartum, when progesterone levels decrease dramatically. In sum, pregnancy-related increases in estrogens may contribute to increases in SDB, whereas progesterone may be protective (Venkata and Venkateshiah, 2009).

To our knowledge, the literature on the correlation between pregnancy and later-life sleep patterns is limited. The only such study we identified examined whether snoring during pregnancy predicted later life SDB and failed to find an association (Chaggar et al., 2021).

#### Links of Sleep and Memory in Pregnancy

Although both sleep disturbances and memory complaints are common among pregnant women, studies examining the links between sleep and memory in this population are scarce. One study found that, among pregnant women in their third trimester, those who reported poorer sleep quality had poorer self-rated memory performance, compared to those who reported good sleep quality (Zhang et al., 2021). The authors speculated that estrogens and progesterone may contribute to poor sleep quality in pregnancy, but did not directly investigate these associations. Another study found lower post-sleep verbal memory performance in women in their third trimester compared to those in their first trimester and non-pregnant women. Whereas women in their third trimester had higher progesterone levels, more fragmented sleep, and lower REM sleep and SWS, no associations were observed between progesterone levels and memory performance, and only weak associations were observed between various sleep parameters and memory performance; the authors noted that findings on the lack of a relationship between sleep and memory must be interpreted with caution due to limited variability in memory performance (Wilson et al., 2013). Unfortunately, the study did not consider correlations between progesterone and sleep. Surprisingly, the authors found that spending less time in SWS and longer time in REM was associated with better post-sleep verbal memory performance, contrary to the notion that SWS promotes sleep-dependent memory consolidation (Wilson et al., 2013). The authors speculated that subtle microstructural changes in sleep architecture not evident when using conventional sleep staging methods or changes in estrogen levels (which were unmeasured) may have obscured a relationship between sleep and memory. Thus, the hypothesis that the greater hormonal concentrations seen in pregnancy affect sleep-dependent memory consolidation cannot be dismissed (Wilson et al., 2013). Furthermore, these sleep problems could extend into the postpartum period. Self-report data from a study comparing recently pregnant, primigravid, and nulliparous women support the idea that women who have been pregnant perceive that their memory is poorer, which may be related to subjective sleep changes (Janes et al., 1999). However, objective memory did not differ between the groups in this study (Janes et al., 1999).

Concentrations of the stress hormone, cortisol, may modulate sleep-dependent memory consolidation during pregnancy. In low concentrations, cortisol appears to be beneficial for sleep-dependent memory consolidation; however higher concentrations, such as those seen in pregnancy, could be detrimental to this process and subsequently affect memory performance (Brunner et al., 2006; Akinloye et al., 2013; Bennion et al., 2015). Given elevated cortisol levels also affect sleep, it is plausible that elevated cortisol and concurrently elevated estradiol and progesterone could produce a synergistic effect to produce larger effects on sleep and memory than they do individually (Smith et al., 2009; Duthie and Reynolds, 2013). However, no study has specifically examined these interactions in pregnancy.

In sum, the extent to which memory and sleep are objectively affected during pregnancy remains unclear. Whereas some studies suggest that memory performance worsens slightly during pregnancy and is accompanied by brain changes that may persist and affect memory long after pregnancy, findings from other studies suggest the biological and environmental experience of pregnancy may protect against brain aging and preserve cognition in later life. Brain reductions seen in pregnancy may be a result of prioritizing maternal behavior and priming the maternal brain for motherhood (Brunton and Russell, 2008). The extent to which sleep, which appears to worsen during pregnancy, plays a role in the link between sex hormones and memory also remains unclear.

### Menopause

The transition into menopause, known as perimenopause, typically begins when women reach their late 40s, and lasts approximately 4 years. Perimenopause is marked by dramatic fluctuations in hormone levels, specifically elvated levels of follicle-stimulating hormone, which usually induce elevated estradiol levels (Santoro et al., 1996). During menopause, however, estrone (E1) becomes the predominant circulating estrogen due to the dramatic decline in ovarian production of estradiol. In post-menopausal women, circulating estradiol is mainly derived from peripheral conversion of androstenedione to estrone *via* aromatase, which is then converted peripherally to estradiol *via* 17β-hydroxysteroid dehydrogenase type 1 (Bulun, 2016). Thus, in postmenopausal women, circulating level of estrone is higher than that of estradiol (Bulun, 2016). Perimenopausal women often experience significant somatic symptoms, including vasomotor symptoms (i.e., “hot flashes”), psychological distress (e.g., depressive symptoms), urogenital symptoms (e.g., frequent urinary tract infections, bladder incontinence), and sleep disturbances. These symptoms result in part from decreased estradiol production, but the presence and extent of these symptoms vary widely. As hormone levels fluctuate, menstrual bleeding becomes increasingly irregular due to an unpredictable pattern of anovulatory (i.e., menstrual cycle without ovulation) and ovulatory cycles. Eventually, the permanent cessation of menses and ovarian estradiol and progesterone production occurs. Menopausal women exhibit low levels of estradiol, which induce a rapid rise in follicle-stimulating hormone and luteinizing hormone levels that gradually decline after peaking a few years after menopause (Jiroutek et al., 1998). The median age of natural menopause is around 51 years (McKinlay et al., 1992). A more detailed and extensive breakdown of the changes that occur with reproductive aging is outlined using the Stages of Reproductive Aging Workshop (STRAW) criteria (Harlow et al., 2012). Unfortunately, most of the studies cited below did not consider these criteria, so we use the three broader categories of premenopause, perimenopause, and post-menopause.

#### Menopause and Memory

As the menopausal transition progresses, some declines are observed in subjective and objective memory measures. Specifically, Weber et al. (2012) found that women in the menopausal transition tend to report lower scores on the Memory Functioning Questionnaire, which assesses self-perceptions of everyday memory performance, and while these subjectively rated scores were associated with objective measures of attention and working memory, they did not correlate with objective verbal memory performance (Weber et al., 2012). Yet other studies suggest that objective verbal memory performance declines during the menopausal transition compared to premenopause and this change is associated with declines in estradiol (Nappi et al., 1999; Maki, 2015). Studies have also demonstrated that earlier onset of menopause is associated with faster cognitive decline later in life (Hogervorst, 2013; Shadyab et al., 2017).

The menopausal transition is associated with decreases in both brain metabolic rate and regional activation, which may be attributable to estradiol depletion (Berent-Spillson et al., 2012; Mosconi et al., 2021). Estradiol and progesterone are thought to protect against hypermetabolism and reduced neuronal mitochondrial function in the hippocampus and prefrontal cortex, both of which have been associated with memory decline in menopause and the initial stages of ADRD (Brann et al., 2012; Picard and McEwen, 2014; Siddiqui et al., 2016; Zárate et al., 2017). However, hormone replacement therapy (HRT) does not consistently demonstrate a neuroprotective benefit (Rapp et al., 2003; Shumaker et al., 2003, 2004; Gleason et al., 2015), which may be related to the timing of HRT in relation to the stages of menopause (Rocca et al., 2011). HRT administered in midlife, prior to perimenopause, has been shown to protect against subsequent cognitive impairment (Whitmer et al., 2011), but HRT appears ineffective in this regard or even increases the risk of dementia when administered post-menopause (Rapp et al., 2003; Shumaker et al., 2003, 2004; Gleason et al., 2015). Thus, there may be a “critical window” in which HRT is effective in reducing cognitive decline and AD risk (Maki, 2013). While exact timing remains controversial, an fMRI study demonstrated higher hippocampal activation and verbal memory when HRT was taken during perimenopause compared to matched controls not taking HRT (Maki et al., 2011). Additionally, the “healthy cell bias of estrogen action” hypothesis suggests that cognitive benefits may be seen only when hormone therapy is administered to non-estrogen-deprived cells (i.e., in premenopausal and perimenopausal women) (Brinton, 2005, 2008). The reduced effects of HRT post-menopause could result from the decreased brain sensitivity to estrogens that may occur as menopause progresses (Pike et al., 2009). Responsiveness of estrogen receptor alpha (ERα) and estrogen receptor beta (Erβ) throughout the brain may decrease with age (Ishunina and Swaab, 2008; Waters et al., 2011; Rettberg et al., 2014), perhaps in response to declining endogenous estradiol levels. For instance, whereas ERβ responds to exogenous estradiol in older rats, ERα does not (Waters et al., 2011). Thus, once the ERα is no longer needed, they may irreversibly stop responding to activation. Taken together, these studies suggest that HRT may best be used to target ERβ specifically or as a preventive rather than therapeutic strategy for memory impairment and ADRD (Brinton, 2005, 2008; Waters et al., 2011).

In sum, while some discrepancy still remains, the decreases in estrogens and progesterone during the menopausal transition seem to be associated with and appear to negatively affect verbal memory. In addition, the timing of HRT has important implications for memory, and may most benefit cognition when initiated shortly after the beginning of menopause.

#### Menopause and Sleep

The menopausal transition is frequently accompanied by sleep disturbances. A meta-analysis comparing peri and postmenopausal women to premenopausal women found an age-independent association between menopausal stages and subjective sleep disturbance (Xu and Lang, 2014). Other studies have found that 40%–70% of women report sleep problems during perimenopause, and 20%–30% of these women continue to experience sleep disturbances even after the menopausal transition (Kravitz et al., 2003; Moline et al., 2003; Dzaja et al., 2005; Schüssler et al., 2008). Thus, decreases in hormone levels may have long-lasting effects on sleep duration and quality. Combined estradiol and cyclic progesterone treatment and estradiol-only treatments have been shown to improve self-reported sleep quality in postmenopausal women with vasomotor symptoms (Cintron et al., 2018; Kagan et al., 2018), and this improvement is not fully mediated by the reduction of vasomotor symptoms (Cintron et al., 2018). Therefore, estradiol and progesterone therapy may be treating menopause-associated sleep disturbances unrelated to vasomotor symptoms. For example, non-micronized progesterone and micronized progesterone decreased wake after sleep onset and increased SWS among post-menopausal women experiencing sleep disturbances (Schüssler et al., 2008; Caufriez et al., 2011). Micronized progesterone has also been found to increase REM sleep in postmenopausal women but only during the first third of the night (Schüssler et al., 2008). A study in older postmenopausal women found that women taking one of two synthetic estrone therapies obtained more SWS compared to women not taking estrogen therapy (Moe et al., 2001). Further, compared to estradiol therapy alone, combined estradiol and various progesterone and progestin therapies produced greater improvements in sleep quality in postmenopausal women (Montplaisir et al., 2001; Saletu, 2003; Gambacciani et al., 2005). Though results of HRT trials suggest that HRT increases SWS, longitudinal observational studies have shown increases in SWS sleep in postmenopausal women not taking HRT, while other metrics suggest that HRT increases wakefulness after sleep onset and sleep fragmentation (Lampio et al., 2017; Kalleinen et al., 2021; Matthews et al., 2021). Further, in one study, postmenopausal and perimenopausal women subjectively reported more sleep complaints than premenopausal women, yet obtained more SWS (Young et al., 2003). Other researchers have suggested the increase in SWS may be compensating for increases in sleep fragmentation and poorer sleep quality induced by hormone decreases and age (Brown and Gervais, 2020), but further research is needed to identify the mechanisms. Lastly, studies have identified a higher risk and severity of SDB during and post menopause, which may be related to decreasing estradiol and progesterone levels (Anttalainen et al., 2006; Polo-Kantola, 2007; Mirer et al., 2017). In summary, decreased estradiol and progesterone levels are associated with greater sleep disturbance in the menopausal transition. Although these sleep disturbances may be alleviated by estradiol and progesterone therapy, mechanisms driving improvement remain to be clarified.

#### Links of Sleep and Memory in Menopause

Both sleep and memory tend to worsen with age, with poorer outcomes among women compared to men (Soares, 2005; Zhang and Wing, 2006; Li and Singh, 2014; Mazure and Swendsen, 2016). Although studies have begun to address the role of sex hormones in the relationship between sleep and memory in older age, it has not been definitively characterized (Gervais et al., 2017; Hajali et al., 2019). A study found that combined estradiol and HRT, consisting of various progestins, did not improve visual episodic memory performance following sleep deprivation in postmenopausal women (Alhola et al., 2005); however, the menopausal stage at which hormone therapy was initiated was not specified. This may be a critical consideration, given the aforementioned importance of HRT timing for cognitive outcomes. Although the effect of endogenous and exogenous hormones on sleep-loss-induced cognitive deficits remains debated, a substantial literature demonstrates that poorer sleep quality and quantity are associated with cognitive impairment in older women (Blackwell et al., 2006; Yaffe et al., 2011; Diem et al., 2016), which we discuss further below.

With respect to the effects of treatment, estradiol valerate and combined estradiol valerate plus progestin improved subjective and objective sleep quality and cognition in postmenopausal women with insomnia (Saletu, 2003). Further, in an RCT of women randomized to either equine estrogen, transdermal 17β-estradiol therapy, or placebo, Zeydan et al. (2021) found better sleep quality at end of the study (measured by the Pittsburgh Sleep Quality Index Global score) was associated with higher visual attention and executive function among women who received equine estrogen or transdermal 17β-estradiol therapy, but not those in the placebo group. Studies are needed to investigate the extent to which estrogens may modify the effects of sleep on cognition and whether improvements in sleep mediate the effect of HRT on cognition (Saletu, 2003; Zeydan et al., 2021).

Importantly, although aging itself is associated with reductions in sleep-spindle density and in the beneficial effects of spindles on memory consolidation, aging-related changes in spindles may differ between men and women. Martin et al. (2013) found that older men had higher spindle frequency than younger and middle-aged men, whereas older women had lower spindle density compared to younger and middle-aged women. Helfrich et al. (2018), however, found lower sleep spindle density and slow oscillation-spindle coupling were associated with lower episodic memory performance in older men and women and that their younger counterparts had higher spindle density and coupling that was linked to better memory performance. In sum, the extent to which sleep affects memory during and post menopause, and the potential interactions of hormonal changes with sleep with respect to cognition, remain to be clarified by further observational and intervention studies across the menopausal transition.

## Discussion

Above, we have reviewed the extensive literature documenting links of female sex hormones with both sleep and memory, and the relatively sparse literature investigating how sex hormones and sleep interrelate with respect to memory across the menstrual cycle, in pregnancy, and menopause. While there is still controversy as to whether fluctuating sex hormones are significant driving factors for changes in memory and sleep, there is considerable literature supporting this hypothesis and multiple avenues for further investigation. Moderately elevated levels of progesterone and estrogens appear beneficial for memory and sleep, while exceptionally high or low levels may negatively affect both. These findings must be considered, however, alongside those from studies finding no significant influences of sex hormones on sleep and memory. In this section, we present candidate models of these complicated interrelationships.

### Female Sex Hormones, Sleep, and Memory

**Figure 1:**
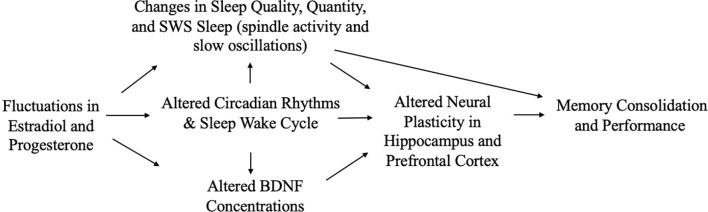
Interrelationships of changes in estradiol and progesterone with sleep, brain function, and memory across the menstrual cycle.

Given the influence of estrogens and progesterone on sleep, and the established links between sleep and memory, sex hormone-induced changes in sleep may mediate associations between sex hormones and memory (Figure 1). For example, alterations in sleep spindle frequency and density and changes in SWS quantity—which are essential for sleep-dependent memory consolidation—may play an important mediating role for memory changes during times of fluctuating estradiol and progesterone levels as observed in different phases of the menstrual cycle (Diekelmann and Born, 2010a; Fogel and Smith, 2011). Both endogenous and exogenous estradiol upregulate progesterone receptors, so estradiol may potentiate progesterone’s effects on sleep spindles; however, more studies are needed in this domain (Bayliss and Millhorn, 1992; Diotel et al., 2011; Brown and Gervais, 2020). Beyond their direct impact on memory consolidation, sleep spindles may also alter longer-term memory outcomes. For example, spindles may enhance hippocampal and prefrontal cortex synaptic plasticity and neurogenesis, both of which are important for next-day memory performance and longer-term maintenance of cognitive health, and are influenced by estrogen and progesterone fluctuations, as described above (Fogel and Smith, 2011; Frankfurt and Luine, 2015; Fernandez and Lüthi, 2020).

**Figure 2:**
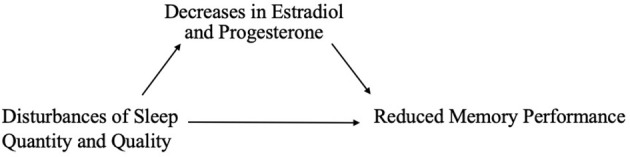
Reduction in sex hormones as a mediator of associations between poor sleep and memory performance.

On the other hand, hormonal changes may mediate the effects of sleep on memory (Figure 2). Carter et al. (2012) found that one night of sleep deprivation decreased progesterone but not estradiol levels in women; however, Lustig et al. (2017) found that female sex hormone levels remained unchanged following sleep deprivation. A bidirectional relationship may also exist, such that sleep disruption reduces estradiol and progesterone, which in turn worsens sleep; this interplay could translate into negative effects on memory (Terán-Pérez et al., 2012).

**Figure 3:**
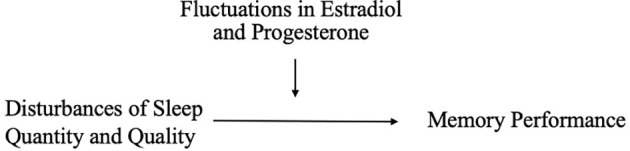
Fluctuations in estradiol and progesterone moderate the relationship between sleep and memory performance.

It is also possible that sex hormones and sleep interact to affect memory (Figure 3). In ovariectomized female rats, REM sleep deprivation more severely impaired spatial memory performance, when compared to REM deprivation in male and intact female rats (Hajali et al., 2012; Saadati et al., 2015). Another study, in which rats were exposed to 72 h of sleep deprivation, rather than just REM sleep deprivation, found similar results (Esmaeilpour et al., 2015). Saadati et al. (2014b) found that 72-h sleep-deprived ovariectomized rats had lower neuronal LTP in the CA1 region of the hippocampus compared to sleep-deprived intact female rats. Further, BDNF levels were reduced in ovariectomized rats compared to intact rats following sleep deprivation, suggesting decreases in BDNF may facilitate a moderating effect of estrogen (Saadati et al., 2014a). Conversely, Hajali et al. (2015) found no differences in LTP in CA-hippocampal neurons or BDNF between ovariectomized rats and intact female rats following sleep deprivation. However, that study did not control for the menstrual phase or fluctuations in hormone levels in the intact rats (Hajali et al., 2015). Thus, more studies, including human studies with objective sleep measures, are needed to characterize inter-relationships among sex hormones, sleep, and memory.

### Implications for AD and Related Dementias (ADRD)

Significant cognitive decline—and decline in memory in particular—is a defining feature of dementia due to AD (World Health Statistics, 2017). Female sex is a prominent known risk factor for AD (Snyder et al., 2016; Nebel et al., 2018; Rahman et al., 2019), and changes in estradiol and progesterone and sleep disturbance all are linked to ADRD (Carroll et al., 2007; Kang et al., 2009; Janicki and Schupf, 2010; Peter-Derex et al., 2015). Scant research, however, has examined the interplay of sex hormones and sleep with regard to ADRD risk.

Several putative neuroprotective mechanisms may link sex hormones to reduced AD risk, including regulation of neuronal mitochondrial function, glial cell function, and Aβ levels (Li et al., 2000; Nilsen et al., 2006; Kaur et al., 2007; Acaz-Fonseca et al., 2014; Hong et al., 2018). For example, in older adults, lower estradiol and progesterone levels are associated with greater Aβ accumulation and neuroinflammation (Vest and Pike, 2013; Zárate et al., 2017), both of which are also affected by sleep (Brown et al., 2016; Pak et al., 2020).

Many studies also link sleep disturbance to clinical ADRD and AD biomarkers (Yaffe et al., 2011; Lim et al., 2013; Chen et al., 2016; Diem et al., 2016; Sabia et al., 2021). Several recent studies have tied insufficient or poor-quality sleep to a greater Aβ burden in late middle-aged and older adults (Ju et al., 2013; Spira et al., 2013; Sprecher et al., 2015; Winer et al., 2020, 2021), and studies in mice, *Drosophila*, and humans suggest a causal role of sleep disturbance in the AD pathophysiological process (Kang et al., 2009; Tabuchi et al., 2015; Shokri-Kojori et al., 2018).

In general, two mechanisms have been posited to explain these sleep-Aβ links, and both may be affected by sex hormone fluctuations that cause or result from sleep disturbances. The first invokes the “glymphatic system,” a waste clearance system mediated by astrocytes that involves the expansion of the brain’s extracellular space, permitting the interface of cerebrospinal fluid (CSF) and interstitial fluid (ISF) to remove toxic metabolites from the brain (Jessen et al., 2015). This clearance process is believed to be most active during SWS (Mendelsohn and Larrick, 2013; Xie et al., 2013; Ju et al., 2017), implicating sleep in Aβ clearance. The second potential mechanism tying sleep loss to AD pathology involves excessive neuronal activation. The synaptic homeostasis hypothesis states that SWS decreases the synaptic strength that accumulates during wakefulness, and that insufficient SWS would therefore prevent this downscaling (Tononi and Cirelli, 2006). Because elevated neuronal activity increases Aβ production (Li et al., 2013), insufficient SWS due to sleep disturbances could thereby promote Aβ deposition, which further disrupts sleep, forming a positive-feedback loop (Ju et al., 2014).

**Figure 4:**
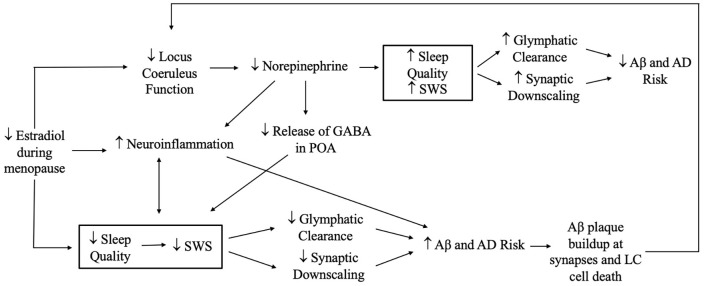
Potential effects of estradiol reduction during menopause on sleep and Alzheimer’s disease (AD) risk. Reductions in estradiol during menopause can theoretically have both beneficial and adverse effects on sleep quality and SWS. Decreases in estradiol can decrease stimulation and function of the locus coeruleus and subsequent norepinephrine production, leading to increases in slow wave sleep (SWS) and enhancements in glymphatic clearance and synaptic downscaling that prevent amyloid beta (Aβ) aggregation and AD risk. On the other hand, reductions in estradiol and norepinephrine could increase neuroinflammation, which promotes sleep disturbances and decreases SWS, leading to further neuroinflammation and forming a bidirectional link with sleep. Reductions in norepinephrine may decrease gamma-aminobutyric acid (GABA) in the preoptic area (POA), thereby increasing the risk of sleep disturbances. Theoretically, declines in estradiol could also degrade sleep quality, decreasing SWS. Subsequent buildup of Aβ could in turn lead to Aβ plaque formation, including at synapses of the LC, and result in LC cell death. The latter would further disrupt locus coeruleus function, creating a feedback loop. Note: not all plausible pathways are shown.

**Figure 5:**
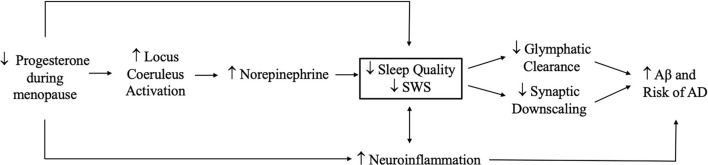
Effects of progesterone reduction during menopause on sleep and Alzheimer’s disease (AD) risk. Reductions in progesterone during menopause may increase activation of the locus coeruleus and subsequent norepinephrine production, leading to decreases in slow wave sleep (SWS) and reductions in glymphatic clearance and synaptic downscaling that promote amyloid beta (Aβ) aggregation and AD risk. Additionally, reductions in progesterone could increase neuroinflammation, which promotes sleep disturbances and lowers SWS, leading to further neuroinflammation and a bidirectional link with sleep. Note: not all plausible pathways are shown.

Hormonal changes during menopause may have effects on sleep, with implications for glymphatic clearance of Aβ and for Aβ production that results from excessive neuronal activity. Estradiol and progesterone are associated with greater SWS during the reproductive years (Baker et al., 2001) and HRT appears to increase SWS in postmenopausal women experiencing sleep disturbances (Moe et al., 2001). The sleep disturbances reported in menopause (Xu and Lang, 2014) may reduce SWS and thereby decrease glymphatic system function and synaptic downscaling, ultimately increasing AD pathology (Kang et al., 2009) (Figures 4 and 5). However studies to date have yielded conflicting findings on sex differences related to the glymphatic system. Giannetto et al. (2020) found no sex differences in glymphatic influx in healthy adult rats, but Duarte et al. (2020) suggested that age, sex, and sex hormones, and circadian rhythms may affect Aβ degradation and subsequent glymphatic clearance, although the mechanisms by which they do so is unclear. To our knowledge, no studies have assessed the effects of estradiol and progesterone on synaptic homeostasis in the context of AD.

Sex differences in norepinephrine production may also adversely affect sleep, with implications for AD pathogenesis in women. Sex differences have been noted in the locus coeruleus (LC), the primary site of norepinephrine synthesis, including larger LC size and higher norepinephrine production in females due to higher estradiol levels (Bangasser et al., 2016). Conversely, progesterone is thought to reduce norepinephrine concentrations and LC activity (Chaudhuri et al., 1992; Lima et al., 2017). Thus, reductions in estradiol and progesterone concentrations in women during and post menopause could, theoretically, modulate LC noradrenergic neuron function with subsequent effects on sleep that could alter glymphatic clearance and Aβ production to promote AD pathology; however, these interrelationships are complex. Menopausal reductions in estradiol and progesterone could, depending on the pathway considered, lead to increases or decreases in sleep quality and SWS. For example, we might expect menopause-related reductions in estradiol to decrease LC activation and norepinephrine concentrations that in turn *increase* SWS and subsequently increase glymphatic clearance and downscaling of neuronal activity (Nedergaard and Goldman, 2016) (Figure 4). On the other hand, reductions in estradiol and norepinephrine could decrease stimulation of GABA release in the POA (Herbison, 1997), and subsequently *decrease* SWS, thereby decreasing glymphatic clearance and synaptic downscaling, and increasing the risk of Aβ aggregation (Figure 4). Further, menopause is characterized by a decrease in progesterone that could in theory increase LC activity and norepinephrine levels which, in turn, *decrease* SWS with implications for AD protein aggregation (Figure 5). Notably, the LC and its noradrenergic neurons are dysfunctional in AD, but the extent to which sleep disturbance contributes to or results from Aβ aggregation in the LC is unclear, as is the extent to which a bi-directional association better accounts for this link (Ju et al., 2014; Ross et al., 2015; Fjell et al., 2018). To our knowledge, no studies have investigated how the depletion of estradiol and progesterone in menopause affects norepinephrine levels, and subsequently sleep, glymphatic flow, and synaptic homeostasis.

Sex hormones and sleep modulate systemic inflammation and neuroinflammation, which play an important role in the AD pathological cascade and in ADRD more broadly (Heneka et al., 2015). Estrogens and progesterone both protect against neuroinflammation (Zárate et al., 2017), and sleep disturbances may increase inflammation in the CNS and periphery and thereby increase the Aβ burden (Kinney et al., 2018; Irwin and Vitiello, 2019). Notably, norepinephrine is also thought to have anti-neuroinflammatory properties, which could be amplified through hormone-mediated mechanisms (Luckey et al., 2021) (Figures 4 and 5). It is unclear whether hormone-induced changes in sleep promote inflammation that in turn promotes the development of ADRD pathology, or if sleep disturbances and hormones interact to exert this effect. For example, disturbed sleep at times marked by specific hormonal changes (e.g., menstrual cycle phases, pregnancy, menopause) may have more pronounced effects on neuroinflammation and resultant ADRD pathophysiology. These pathways are not mutually exclusive, and research on these interrelationships and the role of neuroinflammation in sleep-related glymphatic function and synaptic homeostasis is needed.

To our knowledge, only one study has examined interrelationships among estrogen, sleep changes, and Aβ deposition in ADRD. Specifically, Zeydan et al. (2021) investigated the association between self-reported sleep quality and cortical Aβ deposition in postmenopausal women receiving either oral conjugated equine estrogen or transdermal estradiol replacement therapy. They found that reports of better sleep quality were associated with lower Aβ deposition in the transdermal estradiol group, compared to the oral conjugated equine estrogen group and placebo (Zeydan et al., 2021). Thus, the differences in pharmacokinetics and pharmacodynamics between the type and mode of delivery of estrogen may modify associations between sleep and Aβ deposition.

## Conclusion

While it is established that sex differences exist in sleep and memory, and in the prevalence of ADRD, the exact mechanisms driving these differences remain unclear. The evidence presented in this review suggests that estrogens and progesterone play important roles in altering both sleep and memory performance across the adult female lifespan, and that sleep may act as a mediator of the link between sex hormone fluctuations and memory changes. Moreover, sex hormone fluctuations may mediate or moderate the relationship between sleep and memory changes.

Research in this area, however, is scarce and inconclusive. More studies are clearly needed to untangle the complex relationships among female sex hormones, sleep, and memory. Results could have considerable implications for the optimization of memory across the adult female lifespan, and potentially for the prevention of cognitive decline and dementia. Because changes in sleep and memory performance are seen in times of both upward and downward hormonal fluctuations (i.e., pregnancy and menopause), sleep and memory deficits may not solely be due to *inter-individual* differences in absolute hormone levels. Rather, *intra-individual* deviations from homeostatic hormone levels may negatively affect brain function, sleep, and memory (Baker and Lee, 2018; Brown and Gervais, 2020). Women can undergo decades of hormonally induced fluctuations in sleep architecture, duration, and quality resulting from the menstrual cycle, pregnancy, and menopause. It is unknown, however, how such sleep perturbations—as well as those resulting from child-rearing and other caregiving activities disproportionately affecting women across the life course—affect cognitive trajectories, AD pathology, and dementia, or account for sex differences in ADRD (Gervais et al., 2017; Baker et al., 2019; Brown and Gervais, 2020). Future research should examine if the aggregation of smaller changes in hormonal concentration during the menstrual cycle and more pronounced hormonal changes in pregnancy and menopause could have an additive negative effect on cognition and sleep later in life, and whether there may be multiple time points in a woman’s lifespan for intervention to reduce ADRD risk later in life through interventions targeting hormones, sleep, or both (Brown and Gervais, 2020). Longitudinal observational research, as well as experimental studies in both animals and humans (i.e., clinical trials), are needed to further interrogate associations among female sex hormones, sleep, and memory, investigate relevant mechanisms, and ultimately develop hormone- and sleep-focused therapies to optimize memory performance, and perhaps prevent ADRD.

## Author Contributions

YH, AS, and JP contributed to conception of study topic. YH performed initial literature search and wrote first draft of manuscript. All authors contributed to the article and approved the submitted version.

## Conflict of Interest

AS received payment for serving as a consultant for Merck, and received honoraria from Springer Nature Switzerland AG for guest editing special issues of Current Sleep Medicine Reports. The remaining authors declare that the research was conducted in the absence of any commercial or financial relationships that could be construed as a potential conflict of interest.

## Publisher’s Note

All claims expressed in this article are solely those of the authors and do not necessarily represent those of their affiliated organizations, or those of the publisher, the editors and the reviewers. Any product that may be evaluated in this article, or claim that may be made by its manufacturer, is not guaranteed or endorsed by the publisher.

## References

[B1] Abel T. Havekes R. Saletin J. M. Walker M. P. (2013). Sleep, plasticity and memory from molecules to whole-brain networks. Curr. Biol. 23, R774–R788. 10.1016/j.cub.2013.07.025 24028961 PMC4263505

[B2] Acaz-Fonseca E. Sanchez-Gonzalez R. Azcoitia I. Arevalo M. A. Garcia-Segura L. M. (2014). Role of astrocytes in the neuroprotective actions of 17β-estradiol and selective estrogen receptor modulators. Mol. Cell. Endocrinol. 389, 48–57. 10.1016/j.mce.2014.01.009 24444786

[B3] Aeschbach D. Santhi N. (2013). Attention and memory changes. Encyclopedia Sleep 1, 217–224. 10.1016/B978-0-12-378610-4.00043-7

[B4] Ajayi A. F. Akhigbe R. E. (2020). Staging of the estrous cycle and induction of estrus in experimental rodents: an update. Fertil. Res. Pract. 6:5. 10.1186/s40738-020-00074-3 32190339 PMC7071652

[B5] Akinloye O. Obikoya O. M. Jegede A. I. Oparinde D. P. Arowojolu A. O. (2013). Cortisol plays central role in biochemical changes during pregnancy. Int. J. Med. Biomed. Res. 2, 3–12. 10.14194/ijmbr.212

[B6] Alhola P. Tallus M. Kylmälä M. Portin R. Polo-Kantola P. (2005). Sleep deprivation, cognitive performance and hormone therapy in postmenopausal women. Menopause 12, 149–155. 10.1097/00042192-200512020-00008 15772561

[B7] Almey A. Milner T. A. Brake W. G. (2015). Estrogen receptors in the central nervous system and their implication for dopamine-dependent cognition in females. Horm. Behav. 74, 125–138. 10.1016/j.yhbeh.2015.06.010 26122294 PMC4820286

[B8] Anaclet C. Lin J. S. Vetrivelan R. Krenzer M. Vong L. Fuller P. M.. (2012). Identification and characterization of a sleep-active cell group in the rostral medullary brainstem. J. Neurosci. 32, 17970–17976. 10.1523/JNEUROSCI.0620-12.2012 23238713 PMC3564016

[B9] Andreano J. M. Cahill L. (2009). Sex influences on the neurobiology of learning and memory. Learn. Mem. 16, 248–266. 10.1101/lm.918309 19318467

[B10] Anttalainen U. Saaresranta T. Aittokallio J. Kalleinen N. Vahlberg T. Virtanen I.. (2006). Impact of menopause on the manifestation and severity of sleep-disordered breathing. Acta Obstet. Gynecol. Scand. 85, 1381–1388. 10.1080/00016340600935649 17091421

[B11] Backhaus J. Junghanns K. (2006). Daytime naps improve procedural motor memory. Sleep Med. 7, 508–512. 10.1016/j.sleep.2006.04.002 16931152

[B12] Baghdadi G. Towhidkhah F. Rajabi M. (2021). Neurocognitive Mechanisms of Attention: Computational Models, Physiology and Disease States. Cambridge, MA: Elsevier Academic Press.

[B13] Bailey M. Silver R. (2014). Sex differences in circadian timing systems: implications for disease. Front. Neuroendocrinol. 35, 111–139. 10.1016/j.yfrne.2013.11.003 24287074 PMC4041593

[B14] Bairam A. Uppari N. Mubayed S. Joseph V. (2015). An overview on the respiratory stimulant effects of caffeine and progesterone on response to hypoxia and apnea frequency in developing rats. Adv. Exp. Med. Biol. 860, 211–220. 10.1007/978-3-319-18440-1_23 26303483

[B15] Baker F. C. Driver H. S. (2004). Self-reported sleep across the menstrual cycle in young, healthy women. J. Psychosom. Res. 56, 239–243. 10.1016/S0022-3999(03)00067-9 15016584

[B16] Baker F. C. Lee K. A. (2018). Menstrual cycle effects on sleep. Sleep Med. Clin. 13, 283–294. 10.1016/j.jsmc.2018.04.002 30098748

[B17] Baker F. C. Mitchell D. Driver H. S. (2001). Oral contraceptives alter sleep and raise body temperature in young women. Pflugers Arch. 442, 729–737. 10.1007/s004240100582 11512029

[B18] Baker F. C. Sattari N. de Zambotti M. Goldstone A. Alaynick W. A. Mednick S. C. (2019). Impact of sex steroids and reproductive stage on sleep-dependent memory consolidation in women. Neurobiol. Learn. Mem. 160, 118–131. 10.1016/j.nlm.2018.03.017 29574082 PMC6150846

[B19] Bangasser D. A. Wiersielis K. R. Khantsis S. (2016). Sex differences in the locus coeruleus-norepinephrine system and its regulation by stress. Brain Res. 1641, 177–188. 10.1016/j.brainres.2015.11.021 26607253 PMC4875880

[B20] Banta Lavenex P. Lavenex P. (2010). Spatial relational learning and memory abilities do not differ between men and women in a real-world, open-field environment. Behav. Brain Res. 207, 125–137. 10.1016/j.bbr.2009.09.046 19800920

[B21] Barba-Müller E. Craddock S. Carmona S. Hoekzema E. (2019). Brain plasticity in pregnancy and the postpartum period: links to maternal caregiving and mental health. Arch. Womens Ment. Health 22, 289–299. 10.1007/s00737-018-0889-z 30008085 PMC6440938

[B22] Barker J. M. Galea L. A. (2008). Repeated estradiol administration alters different aspects of neurogenesis and cell death in the hippocampus of female, but not male, rats. Neuroscience 152, 888–902. 10.1016/j.neuroscience.2007.10.071 18353559

[B23] Barth C. Steele C. J. Mueller K. Rekkas V. P. Arélin K. Pampel A.. (2016). *In-vivo* dynamics of the human hippocampus across the menstrual cycle. Sci. Rep. 6:32833. 10.1038/srep32833 27713470 PMC5054394

[B24] Bayliss D. A. Millhorn D. E. (1992). Central neural mechanisms of progesterone action: application to the respiratory system. J. Appl. Physiol. (1985) 73, 393–404. 10.1152/jappl.1992.73.2.393 1399957

[B25] Begliuomini S. Casarosa E. Pluchino N. Lenzi E. Centofanti M. Freschi L.. (2007). Influence of endogenous and exogenous sex hormones on plasma brain-derived neurotrophic factor. Hum. Reprod. 22, 995–1002. 10.1093/humrep/del479 17251358

[B26] Bennion K. A. Mickley Steinmetz K. R. Kensinger E. A. Payne J. D. (2015). Sleep and cortisol interact to support memory consolidation. Cereb. Cortex 25, 646–657. 10.1093/cercor/bht255 24072888

[B27] Berent-Spillson A. Persad C. C. Love T. Sowers M. Randolph J. F. Zubieta J. K.. (2012). Hormonal environment affects cognition independent of age during the menopause transition. J. Clin. Endocrinol. Metabol. 97, E1686–E1694. 10.1210/jc.2012-1365 22730514 PMC3431577

[B28] Berry R. B. Brooks R. Gamaldo C. E. Harding S. M. Lloyd R. M. Marcus C. L.. (2015). The AASM Manual for the Scoring of Sleep and Associated Events: Rules, Terminology and Technical Specifications, Version 2.2. Darien, IL: American Academy of Sleep Medicine. Available online at: www.aasmnet.org.

[B29] Blackwell T. Yaffe K. Ancoli-Israel S. Schneider J. L. Cauley J. A. Hillier T. A.. (2006). Poor sleep is associated with impaired cognitive function in older women: the study of osteoporotic fractures. J. Gerontol. A Biol. Sci. Med. Sci. 61, 405–410. 10.1093/gerona/61.4.405 16611709

[B30] Born J. (2010). Slow-wave sleep and the consolidation of long-term memory. World J. Biol. Psychiatry 11, 16–21. 10.3109/15622971003637637 20509828

[B31] Brann D. Raz L. Wang R. Vadlamudi R. Zhang Q. (2012). Oestrogen signalling and neuroprotection in cerebral ischaemia. J. Neuroendocrinol. 24, 34–47. 10.1111/j.1365-2826.2011.02185.x 21722216 PMC3324183

[B32] Brett M. Baxendale S. (2001). Motherhood and memory: a review. Psychoneuroendocrinology 26, 339–362. 10.1016/s0306-4530(01)00003-8 11259856

[B33] Brindle P. M. Brown M. W. Brown J. Griffith H. B. Turner G. M. (1991). Objective and subjective memory impairment in pregnancy. Psychol. Med. 21, 647–653. 10.1017/s0033291700022285 1946853

[B34] Brinton R. D. (2005). Investigative models for determining hormone therapy-induced outcomes in brain: evidence in support of a healthy cell bias of estrogen action. Ann. N Y Acad. Sci. 1052, 57–74. 10.1196/annals.1347.005 16024751

[B35] Brinton R. D. (2008). The healthy cell bias of estrogen action: mitochondrial bioenergetics and neurological implications. Trends Neurosci. 31, 529–537. 10.1016/j.tins.2008.07.003 18774188 PMC10124615

[B36] Brown A. Gervais N. J. (2020). Role of ovarian hormones in the modulation of sleep in females across the adult lifespan. Endocrinology 161:bQ19a128. 10.1210/endocr/bQ19a128 32735650 PMC7450669

[B37] Brown B. M. Rainey-Smith S. R. Villemagne V. L. Weinborn M. Bucks R. S. Sohrabi H. R.. (2016). The relationship between sleep quality and brain amyloid burden. Sleep 39, 1063–1068. 10.5665/sleep.5756 27091528 PMC4835305

[B38] Brown E. Schaffir J. (2019). “Pregnancy brain”: a review of cognitive changes in pregnancy and postpartum. Obstet. Gynecol. Surv. 74, 178–185. 10.1097/OGX.0000000000000655 31634921

[B39] Brownell L. G. West P. Kryger M. H. (1986). Breathing during sleep in normal pregnant women. Am. Rev. Respir. Dis. 133, 38–41. 10.1164/arrd.1986.133.1.38 3942378

[B40] Brunner D. P. Münch M. Biedermann K. Huch R. Huch A. Borbély A. A. (1994). Changes in sleep and sleep electroencephalogram during pregnancy. Sleep 17, 576–582. 10.1093/sleep/17.7.576 7846455

[B41] Brunner R. Schaefer D. Hess K. Parzer P. Resch F. Schwab S. (2006). Effect of high-dose cortisol on memory functions. Ann. N Y Acad. Sci. 1071, 434–437. 10.1196/annals.1364.037 16891593

[B42] Brunton P. J. Russell J. A. (2008). The expectant brain: adapting for motherhood. Nat. Rev. Neurosci. 9, 11–25. 10.1038/nrn2280 18073776

[B43] Bulun S. (2016). “Chapter 17-Physiology and pathology of the female reproductive axis,” in Williams Textbook of Endocrinology (Thirteenth edition), 589–663. 10.1016/b978-0-323-29738-7.00017-4

[B44] Cabrera-Pedraza V. R. de Jesús Gómez-Villalobos M. de la Cruz F. Aguilar-Alonso P. Zamudio S. Flores G. (2017). Pregnancy improves cognitive deficit and neuronal morphology atrophy in the prefrontal cortex and hippocampus of aging spontaneously hypertensive rats. Synapse 71:e21991. 10.1002/syn.21991 28681457

[B45] Camina E. Güell F. (2017). The neuroanatomical, neurophysiological and psychological basis of memory: current models and their origins. Front. Pharmacol. 8:438. 10.3389/fphar.2017.00438 28713278 PMC5491610

[B46] Carrier J. Semba K. Deurveilher S. Drogos L. Cyr-Cronier J. Lord C.. (2017). Sex differences in age-related changes in the sleep-wake cycle. Front. Neuroendocrinol. 47, 66–85. 10.1016/j.yfrne.2017.07.004 28757114

[B47] Carroll J. C. Rosario E. R. Chang L. Stanczyk F. Z. Oddo S. LaFerla F. M.. (2007). Progesterone and estrogen regulate Alzheimer-like neuropathology in female 3xTg-AD mice. J. Neurosci. 27, 13357–13365. 10.1523/JNEUROSCI.2718-07.2007 18045930 PMC6673397

[B48] Carskadon M. A. Dement W. C. (2011). “Monitoring and staging human sleep,” in Principles and practice of sleep medicine, 5th edition, Kryger M. H. Roth T. Dement W. C. (St. Louis: Elsevier Saunders), 16–26.

[B49] Carter J. R. Durocher J. J. Larson R. A. DellaValla J. P. Yang H. (2012). Sympathetic neural responses to 24-hour sleep deprivation in humans: sex differences. Am. J. Physiol. Heart Circ. Physiol. 302, H1991–H1997. 10.1152/ajpheart.01132.2011 22408018 PMC3362107

[B50] Caufriez A. Leproult R. L’Hermite-Balériaux M. Kerkhofs M. Copinschi G. (2011). Progesterone prevents sleep disturbances and modulates GH, TSH and melatonin secretion in postmenopausal women. J. Clin. Endocrinol. Metabol. 96, E614–E623. 10.1210/jc.2010-2558 21289261

[B51] Cauley J. A. (2012). “The demography of aging,” in The Epidemiology of Aging, eds Newman A. B. Cauley J. A. (Dordrecht: Springer), 3–14.

[B52] Chaggar G. Sutherland K. Han F. Fietze I. Penzel T. Benediktsdóttir B.. (2021). Is snoring during pregnancy a predictor of later life obstructive sleep apnoea? A case-control study. Sleep Med. 79, 190–194. 10.1016/j.sleep.2020.10.023 33279414

[B53] Chamberlin N. L. (2004). Functional organization of the parabrachial complex and intertrigeminal region in the control of breathing. Respir. Physiol. Neurobiol. 143, 115–125. 10.1016/j.resp.2004.03.015 15519549

[B54] Chan R. W. Ho L. C. Zhou I. Y. Gao P. P. Chan K. C. Wu E. X. (2015). Structural and functional brain remodeling during pregnancy with diffusion tensor MRI and resting-state functional MRI. PLoS One 10:e0144328. 10.1371/journal.pone.0144328 26658306 PMC4675543

[B55] Chaudhuri S. K. Chattopadhyay R. N. Maitra S. K. Ray S. Chaudhuri S. (1992). Effects of progesterone on some brain neurotransmitters in intact rats. Indian J. Physiol. Pharmacol. 36, 255–258. 1363321

[B56] Cheke L. G. Clayton N. S. (2013). Do different tests of episodic memory produce consistent results in human adults? Learn. Mem. 20, 491–498. 10.1101/lm.030502.113 23955172

[B57] Chen J. C. Espeland M. A. Brunner R. L. Lovato L. C. Wallace R. B. Leng X.. (2016). Sleep duration, cognitive decline and dementia risk in older women. Alzheimers Dement. 12, 21–33. 10.1016/j.jalz.2015.03.004 26086180 PMC4679723

[B58] Chokroverty S. (2017). “Overview of normal sleep,” in Sleep Disorders Medicine, ed Chokroverty S. (New York, NY: Springer), 5–27.

[B59] Cintron D. Lahr B. D. Bailey K. R. Santoro N. Lloyd R. Manson J. E.. (2018). Effects of oral versus transdermal menopausal hormone treatments on self-reported sleep domains and their association with vasomotor symptoms in recently menopausal women enrolled in the Kronos Early Estrogen Prevention Study (KEEPS). Menopause 25, 145–153. 10.1097/GME.0000000000000971 28832429 PMC5771895

[B60] Cox R. Hofman W. F. Talamini L. M. (2012). Involvement of spindles in memory consolidation is slow wave sleep-specific. Learn. Mem. 19, 264–267. 10.1101/lm.026252.112 22700468

[B61] Craig M. C. Fletcher P. C. Daly E. M. Rymer J. Brammer M. Giampietro V.. (2008). Physiological variation in estradiol and brain function: a functional magnetic resonance imaging study of verbal memory across the follicular phase of the menstrual cycle. Horm. Behav. 53, 503–508. 10.1016/j.yhbeh.2007.11.005 18279872

[B62] Crawley R. Grant S. Hinshaw K. I. M. (2008). Cognitive changes in pregnancy: mild decline or societal stereotype? Appl. Cogn. Psychol. 22, 1142–1162. 10.1002/acp.1427

[B63] Curran-Rauhut M. A. Petersen S. L. (2002). The distribution of progestin receptor mRNA in the rat brainstem. Brain Res. Gene Expr. Patterns 1, 151–157. 10.1016/s1567-133x(02)00011-x 12638125

[B64] Davies S. J. Lum J. A. Skouteris H. Byrne L. K. Hayden M. J. (2018). Cognitive impairment during pregnancy: a meta-analysis. Med. J. Aust. 208, 35–40. 10.5694/mja17.00131 29320671

[B65] de Lange A. M. G. Barth C. Kaufmann T. Anatürk M. Suri S. Ebmeier K. P.. (2020). The maternal brain: region-specific patterns of brain aging are traceable decades after childbirth. Hum. Brain Mapp. 41, 4718–4729. 10.1002/hbm.25152 32767637 PMC7555081

[B66] De Zeeuw C. I. Ten Brinke M. M. (2015). Motor learning and the cerebellum. Cold Spring Harb. Perspect. Biol. 7:a021683. 10.1101/cshperspect.a021683 26330521 PMC4563713

[B67] Deurveilher S. Rusak B. Semba K. (2009). Estradiol and progesterone modulate spontaneous sleep patterns and recovery from sleep deprivation in ovariectomized rats. Sleep 32, 865–877. 10.1093/SLEEP/32.7.865 19639749 PMC2704917

[B68] Dib R. Gervais N. J. Mongrain V. (2021). A review of the current state of knowledge on sex differences in sleep and circadian phenotypes in rodents. Neurobiol. Sleep Circadian Rhythms 11:100068. 10.1016/j.nbscr.2021.100068 34195482 PMC8240025

[B69] Dickerson B. C. Eichenbaum H. (2010). The episodic memory system: neurocircuitry and disorders. Neuropsychopharmacology 35, 86–104. 10.1038/npp.2009.126 19776728 PMC2882963

[B70] Diekelmann S. Born J. (2010a). Slow-wave sleep takes the leading role in memory reorganization. Nat. Rev. Neurosci. 11:218. 10.1038/nrn2762-c220168316

[B71] Diekelmann S. Born J. (2010b). The memory function of sleep. Nat. Rev. Neurosci. 11, 114–126. 10.1038/nrn2762 20046194

[B72] Diem S. J. Blackwell T. L. Stone K. L. Yaffe K. Tranah G. Cauley J. A.. (2016). Measures of sleep-wake patterns and risk of mild cognitive impairment or dementia in older women. Am. J. Geriatr. Psychiatry 24, 248–258. 10.1016/j.jagp.2015.12.002 26964485 PMC4807599

[B73] Dietrich T. Krings T. Neulen J. Willmes K. Erberich S. Thron A.. (2001). Effects of blood estrogen level on cortical activation patterns during cognitive activation as measured by functional MRI. NeuroImage 13, 425–432. 10.1006/nimg.2001.0703 11170808

[B74] Diotel N. Servili A. Gueguen M. M. Mironov S. Pellegrini E. Vaillant C.. (2011). Nuclear progesterone receptors are up-regulated by estrogens in neurons and radial glial progenitors in the brain of zebrafish. PLoS One 6:e28375. 10.1371/journal.pone.0028375 22140581 PMC3227669

[B76] Driver H. S. Dijk D. J. Werth E. Biedermann K. Borbély A. A. (1996). Sleep and the sleep electroencephalogram across the menstrual cycle in young healthy women. J. Clin. Endocrinol. Metabol. 81, 728–735. 10.1210/jcem.81.2.8636295 8636295

[B77] Driver H. S. McLean H. Kumar D. V. Farr N. Day A. G. Fitzpatrick M. F. (2005). The influence of the menstrual cycle on upper airway resistance and breathing during sleep. Sleep 28, 449–456. 10.1093/sleep/28.4.449 16171289

[B75] Driver H. S. Shapiro C. M. (1992). A longitudinal study of sleep stages in young women during pregnancy and postpartum. Sleep 15, 449–453. 10.1093/sleep/15.5.449 1455129

[B78] Duarte A. C. Furtado A. Hrynchak M. V. Costa A. R. Talhada D. Gonçalves I.. (2020). Age, sex hormones and circadian rhythm regulate the expression of amyloid-β scavengers at the choroid plexus. Int. J. Mol. Sci. 21:6813. 10.3390/ijms21186813 32957439 PMC7554684

[B79] Duthie L. Reynolds R. M. (2013). Changes in the maternal hypothalamic-pituitary-adrenal axis in pregnancy and postpartum: influences on maternal and fetal outcomes. Neuroendocrinology 98, 106–115. 10.1159/000354702 23969897

[B80] Dzaja A. Arber S. Hislop J. Kerkhofs M. Kopp C. Pollmächer T.. (2005). Women’s sleep in health and disease. J. Psychiatr. Res. 39, 55–76. 10.1016/j.jpsychires.2004.05.008 15504424

[B81] Eid R. S. Chaiton J. A. Lieblich S. E. Bodnar T. S. Weinberg J. Galea L. (2019). Early and late effects of maternal experience on hippocampal neurogenesis, microglia and the circulating cytokine milieu. Neurobiol. Aging 78, 1–17. 10.1016/j.neurobiolaging.2019.01.021 30825663

[B82] Elsesser K. (2020). The Myth of Biological Sex. Available online at: https://www.forbes.com/sites/kimelsesser/2020/06/15/the-myth-of-biological-sex/?sh=6c57f7fb76b9. Accessed September 28, 2021.

[B83] Ertman N. Andreano J. M. Cahill L. (2011). Progesterone at encoding predicts subsequent emotional memory. Learn. Mem. 18, 759–763. 10.1101/lm.023267.111 22101178 PMC3222892

[B84] Esmaeilpour K. Sheibani V. Saadati H. (2015). Caffeine improved spatial learning and memory deficit in sleep deprived female rat. Physiol. Pharmacol. 19, 121–129. 10.1016/j.physbeh.2014.10.006 25447468

[B85] Facco F. L. Kramer J. Ho K. H. Zee P. C. Grobman W. A. (2010). Sleep disturbances in pregnancy. Obstet. Gynecol. 115, 77–83. 10.1097/AOG.0b013e3181c4f8ec 20027038

[B86] Facco F. L. Ouyang D. W. Zee P. C. Grobman W. A. (2014). Sleep disordered breathing in a high-risk cohort prevalence and severity across pregnancy. Am. J. Perinatol. 31, 899–904. 10.1055/s-0033-1363768 24515622 PMC4531051

[B87] Fernandez L. M. Lüthi A. (2020). Sleep spindles: mechanisms and functions. Physiol. Rev. 100, 805–868. 10.1152/physrev.00042.2018 31804897

[B88] Fjell A. M. Idland A. V. Sala-Llonch R. Watne L. O. Borza T. Brækhus A.. (2018). Neuroinflammation and tau interact with amyloid in predicting sleep problems in aging independently of atrophy. Cereb. cortex 28, 2775–2785. 10.1093/cercor/bhx157 28655157

[B89] Fogel S. M. Smith C. T. (2011). The function of the sleep spindle: a physiological index of intelligence and a mechanism for sleep-dependent memory consolidation. Neurosci. Biobehav. Rev. 35, 1154–1165. 10.1016/j.neubiorev.2010.12.003 21167865

[B90] Foley D. Ancoli-Israel S. Britz P. Walsh J. (2004). Sleep disturbances and chronic disease in older adults: results of the 2003 National Sleep Foundation Sleep in America Survey. J. Psychosom. Res. 56, 497–502. 10.1016/j.jpsychores.2004.02.010 15172205

[B91] Fox M. Berzuini C. Knapp L. A. Glynn L. M. (2018). Women’s pregnancy life history and Alzheimer’s risk: Can immunoregulation explain the link? Am. J. Alzheimers Dis. Other Demen. 33, 516–526. 10.1177/1533317518786447 30060670 PMC6442681

[B92] Foy M. R. Akopian G. Thompson R. F. (2008). Progesterone regulation of synaptic transmission and plasticity in rodent hippocampus. Learn. Mem. 15, 820–822. 10.1101/lm.1124708 18984562 PMC2632810

[B93] Foy M. R. Baudry M. Akopian G. K. Thompson R. F. (2010). Regulation of hippocampal synaptic plasticity by estrogen and progesterone. Vitam. Horm. 82, 219–239. 10.1016/S0083-6729(10)82012-6 20472141

[B94] Foy M. R. Xu J. Xie X. Brinton R. D. Thompson R. F. Berger T. W. (1999). 17β-estradiol enhances NMDA receptor-mediated EPSPs and long-term potentiation. J. Neurophysiol. 81, 925–929. 10.1152/jn.1999.81.2.925 10036289

[B95] Frankfurt M. Luine V. (2015). The evolving role of dendritic spines and memory: interaction(s) with estradiol. Horm. Behav. 74, 28–36. 10.1016/j.yhbeh.2015.05.004 25993604 PMC4573337

[B96] Fukuda N. Honma H. Kohsaka M. Kobayashi R. Sakakibara S. Kohsaka S.. (1999). Gender difference of slow wave sleep in middle aged and elderly subjects. Psychiatry Clin. Neurosci. 53, 151–153. 10.1046/j.1440-1819.1999.00508.x 10459675

[B97] Galea L. A. Ormerod B. K. Sampath S. Kostaras X. Wilkie D. M. Phelps M. T. (2000). Spatial working memory and hippocampal size across pregnancy in rats. Horm. Behav. 37, 86–95. 10.1006/hbeh.1999.1560 10712861

[B98] Gambacciani M. Ciaponi M. Cappagli B. Monteleone P. Benussi C. Bevilacqua G.. (2005). Effects of low-dose, continuous combined hormone replacement therapy on sleep in symptomatic postmenopausal women. Maturitas 50, 91–97. 10.1016/j.maturitas.2004.04.006 15653005

[B99] Gatewood J. D. Morgan M. D. Eaton M. McNamara I. M. Stevens L. F. Macbeth A. H.. (2005). Motherhood mitigates aging-related decrements in learning and memory and positively affects brain aging in the rat. Brain Res. Bull. 66, 91–98. 10.1016/j.brainresbull.2005.03.016 15982524

[B100] Genzel L. Kiefer T. Renner L. Wehrle R. Kluge M. Grözinger M.. (2012). Sex and modulatory menstrual cycle effects on sleep related memory consolidation. Psychoneuroendocrinology 37, 987–998. 10.1016/j.psyneuen.2011.11.006 22153362

[B101] Gervais N. J. Mong J. A. Lacreuse A. (2017). Ovarian hormones, sleep and cognition across the adult female lifespan: an integrated perspective. Front. Neuroendocrinol. 47, 134–153. 10.1016/j.yfrne.2017.08.002 28803147 PMC7597864

[B102] Giannetto M. Xia M. Stæger F. F. Metcalfe T. Vinitsky H. S. Dang J. A.. (2020). Biological sex does not predict glymphatic influx in healthy young, middle aged or old mice. Sci. Rep. 10:16073. 10.1038/s41598-020-72621-3 32999319 PMC7528110

[B103] Gleason C. E. Dowling N. M. Wharton W. Manson J. E. Miller V. M. Atwood C. S.. (2015). Effects of hormone therapy on cognition and mood in recently postmenopausal women: findings from the randomized, controlled KEEPS-cognitive and affective study. PLoS Med. 12:e1001833. 10.1371/journal.pmed.1001833 26035291 PMC4452757

[B104] Glynn L. M. (2010). Giving birth to a new brain: hormone exposures of pregnancy influence human memory. Psychoneuroendocrinology 35, 1148–1155. 10.1016/j.psyneuen.2010.01.015 20304563

[B105] Gottesmann C. (2002). GABA mechanisms and sleep. Neuroscience 111, 231–239. 10.1016/s0306-4522(02)00034-9 11983310

[B106] Guida M. Rega A. Vivone I. Saccone G. Sarno L. Di Carlo C.. (2020). Variations in sleep associated with different types of hormonal contraceptives. Gynecol. Endocrinol. 36, 166–170. 10.1080/09513590.2019.1640204 31311352

[B107] Hadjimarkou M. M. Benham R. Schwarz J. M. Holder M. K. Mong J. A. (2008). Estradiol suppresses rapid eye movement sleep and activation of sleep-active neurons in the ventrolateral preoptic area. Eur. J. Neurosci. 27, 1780–1792. 10.1111/j.1460-9568.2008.06142.x 18371078

[B108] Hagemann G. Ugur T. Schleussner E. Mentzel H. J. Fitzek C. Witte O. W.. (2011). Changes in brain size during the menstrual cycle. PLoS One 6:e14655. 10.1371/journal.pone.0014655 21326603 PMC3033889

[B109] Hajali V. Andersen M. L. Negah S. S. Sheibani V. (2019). Sex differences in sleep and sleep loss-induced cognitive deficits: the influence of gonadal hormones. Horm. Behav. 108, 50–61. 10.1016/j.yhbeh.2018.12.013 30597139

[B110] Hajali V. Sheibani V. Esmaeili-Mahani S. Shabani M. (2012). Female rats are more susceptible to the deleterious effects of paradoxical sleep deprivation on cognitive performance. Behav. Brain Res. 228, 311–318. 10.1016/j.bbr.2011.12.008 22192378

[B111] Hajali V. Sheibani V. Ghazvini H. Ghadiri T. Valizadeh T. Saadati H.. (2015). Effect of castration on the susceptibility of male rats to the sleep deprivation-induced impairment of behavioral and synaptic plasticity. Neurobiol. Learn. Mem. 123, 140–148. 10.1016/j.nlm.2015.05.008 26079215

[B112] Hampson E. (2018). Estrogens, aging and working memory. Curr. Psychiatry Rep. 20:109. 10.1007/s11920-018-0972-1 30306352 PMC6182645

[B113] Hampson E. (2020). A brief guide to the menstrual cycle and oral contraceptive use for researchers in behavioral endocrinology. Horm. Behav. 119:104655. 10.1016/j.yhbeh.2019.104655 31843564

[B114] Hampson E. (1990a). Estrogen-related variations in human spatial and articulatory-motor skills. Psychoneuroendocrinology 15, 97–111. 10.1016/0306-4530(90)90018-5 2359813

[B115] Hampson E. (1990b). Variations in sex-related cognitive abilities across the menstrual cycle. Brain Cogn. 14, 26–43. 10.1016/0278-2626(90)90058-v 2223043

[B116] Hamson D. K. Roes M. M. Galea L. A. (2016). Sex hormones and cognition: neuroendocrine influences on memory and learning. Compr. Physiol. 6, 1295–1337. 10.1002/cphy.c150031 27347894

[B117] Hao J. Rapp P. R. Leffler A. E. Leffler S. R. Janssen W. G. Lou W.. (2006). Estrogen alters spine number and morphology in prefrontal cortex of aged female rhesus monkeys. J. Neurosci. 26, 2571–2578. 10.1523/JNEUROSCI.3440-05.2006 16510735 PMC6793646

[B118] Harlow S. D. Gass M. Hall J. E. Lobo R. Maki P. Rebar R. W.. (2012). Executive summary of the stages of reproductive aging workshop +10: addressing the unfinished agenda of staging reproductive aging. Climacteric 15, 105–114. 10.3109/13697137.2011.650656 22338612 PMC3580996

[B119] Hastings M. H. Maywood E. S. Brancaccio M. (2018). Generation of circadian rhythms in the suprachiasmatic nucleus. Nat. Rev. Neurosci. 19, 453–469. 10.1038/s41583-018-0026-z 29934559

[B120] Hausmann M. Slabbekoorn D. Van Goozen S. H. Cohen-Kettenis P. T. Güntürkün O. (2000). Sex hormones affect spatial abilities during the menstrual cycle. Behav. Neurosci. 114, 1245–1250. 10.1037//0735-7044.114.6.1245 11142657

[B121] Helfrich R. F. Mander B. A. Jagust W. J. Knight R. T. Walker M. P. (2018). Old brains come uncoupled in sleep: slow wave-spindle synchrony, brain atrophy and forgetting. Neuron 97, 221–230.e4. 10.1016/j.neuron.2017.11.020 29249289 PMC5754239

[B122] Heneka M. T. Carson M. J. El Khoury J. Landreth G. E. Brosseron F. Feinstein D. L.. (2015). Neuroinflammation in Alzheimer’s disease. Lancet Neurol. 14, 388–405. 10.1016/S1474-4422(15)70016-5 25792098 PMC5909703

[B123] Henry J. D. Rendell P. G. (2007). A review of the impact of pregnancy on memory function. J. Clin. Exp. Neuropsychol. 29, 793–803. 10.1080/13803390701612209 18030631

[B124] Herbison A. E. (1997). Estrogen regulation of GABA transmission in rat preoptic area. Brain Res. Bull. 44, 321–326. 10.1016/s0361-9230(97)00210-4 9370195

[B125] Herlitz A. Rehnman J. (2008). Sex differences in episodic memory. Curr. Direct. Psychol. Sci. 17, 52–56. 10.1111/j.1467-8721.2008.00547.x

[B126] Hertz G. Fast A. Feinsilver S. H. Albertario C. L. Schulman H. Fein A. M. (1992). Sleep in normal late pregnancy. Sleep 15, 246–251. 10.1093/sleep/15.3.246 1621025

[B127] Hill R. A. Chua H. K. Jones M. E. Simpson E. R. Boon W. C. (2009). Estrogen deficiency results in apoptosis in the frontal cortex of adult female aromatase knockout mice. Mol. Cell. Neurosci. 41, 1–7. 10.1016/j.mcn.2008.12.009 19185610

[B128] Hoekzema E. Barba-Müller E. Pozzobon C. Picado M. Lucco F. García-García D.. (2017). Pregnancy leads to long-lasting changes in human brain structure. Nat. Neurosci. 20, 287–296. 10.1038/nn.4458 27991897

[B129] Hogervorst E. (2013). Effects of gonadal hormones on cognitive behaviour in elderly men and women. J. Neuroendocrinol. 25, 1182–1195. 10.1111/jne.12080 23895362

[B130] Holth J. K. Fritschi S. K. Wang C. Pedersen N. P. Cirrito J. R. Mahan T. E.. (2019). The sleep-wake cycle regulates brain interstitial fluid tau in mice and CSF tau in humans. Science 363, 880–884. 10.1126/science.aav2546 30679382 PMC6410369

[B131] Hong Y. Liu Y. Zhang G. Wu H. Hou Y. (2018). Progesterone suppresses Aβ42-induced neuroinflammation by enhancing autophagy in astrocytes. Int. Immunopharmacol. 54, 336–343. 10.1016/j.intimp.2017.11.044 29197800

[B132] Huupponen E. Himanen S. L. Värri A. Hasan J. Lehtokangas M. Saarinen J. (2002). A study on gender and age differences in sleep spindles. Neuropsychobiology 45, 99–105. 10.1159/000048684 11893867

[B133] Hyer M. M. Phillips L. L. Neigh G. N. (2018). Sex differences in synaptic plasticity: hormones and beyond. Front. Mol. Neurosci. 11:266. 10.3389/fnmol.2018.00266 30108482 PMC6079238

[B134] Iber C. Ancoli-Israel S. Chesson A. L. Quan S. F. (2007). The AASM Manual for the Scoring of Sleep and Associated Events: Rules, Terminology and Technical Specifications, 1st Edn. Westchester, IL: American Academy of Sleep Medicine.

[B135] Irwin M. R. Vitiello M. V. (2019). Implications of sleep disturbance and inflammation for Alzheimer’s disease dementia. Lancet Neurol. 18, 296–306. 10.1016/S1474-4422(18)30450-2 30661858

[B136] Ishizuka Y. Pollak C. P. Shirakawa S. Kakuma T. Azumi K. USUI A.. (1994). Sleep spindle frequency changes during the menstrual cycle. J. Sleep Res. 3, 26–29. 10.1111/j.1365-2869.1994.tb00100.x 10607105

[B137] Ishunina T. A. Swaab D. F. (2008). Estrogen receptor-α splice variants in the human brain. Gynecol. Endocrinol. 24, 93–98. 10.1080/09513590701705148 18210333

[B138] Janes C. Casey P. Huntsdale C. Angus G. (1999). Memory in pregnancy. I: subjective experiences and objective assessment of implicit, explicit and working memory in primigravid and primiparous women. J. Psychosom. Obstet. Gynecol. 20, 80–87. 10.3109/01674829909075580 10422039

[B139] Janicki S. C. Schupf N. (2010). Hormonal influences on cognition and risk for Alzheimer’s disease. Curr. Neurol. Neurosci. Rep. 10, 359–366. 10.1007/s11910-010-0122-6 20535591 PMC3058507

[B140] Jessen N. A. Munk A. S. Lundgaard I. Nedergaard M. (2015). The glymphatic system: a beginner’s guide. Neurochem. Res. 40, 2583–2599. 10.1007/s11064-015-1581-6 25947369 PMC4636982

[B141] Jiroutek M. R. Chen M. H. Johnston C. C. Longcope C. (1998). Changes in reproductive hormones and sex hormone-binding globulin in a group of postmenopausal women measured over 10 years. Menopause 5, 90–94. 9689202

[B142] Ju Y. E. S. Lucey B. P. Holtzman D. M. (2014). Sleep and Alzheimer disease pathology—a bidirectional relationship. Nat. Rev. Neurol. 10, 115–119. 10.1038/nrneurol.2013.269 24366271 PMC3979317

[B143] Ju Y. E. S. McLeland J. S. Toedebusch C. D. Xiong C. Fagan A. M. Duntley S. P.. (2013). Sleep quality and preclinical Alzheimer disease. JAMA Neurol. 70, 587–593. 10.1001/jamaneurol.2013.2334 23479184 PMC3676720

[B144] Ju Y. E. S. Ooms S. J. Sutphen C. Macauley S. L. Zangrilli M. A. Jerome G.. (2017). Slow wave sleep disruption increases cerebrospinal fluid amyloid-β levels. Brain 140, 2104–2111. 10.1093/brain/awx148 28899014 PMC5790144

[B145] Juraska J. M. (1991). Sex differences in “cognitive” regions of the rat brain. Psychoneuroendocrinology 16, 105–109. 10.1016/0306-4530(91)90073-3 1961834

[B146] Kagan R. Constantine G. Kaunitz A. M. Bernick B. Mirkin S. (2018). Improvement in sleep outcomes with a 17β-estradiol-progesterone oral capsule (TX-001HR) for postmenopausal women. Menopause 26, 622–628. 10.1097/GME.0000000000001278 30586005 PMC6553506

[B147] Kalleinen N. Aittokallio J. Lampio L. Kaisti M. Polo-Kantola P. Polo O.. (2021). Sleep during menopausal transition: a 10-year follow-up. Sleep 44:zsaa283. 10.1093/sleep/zsaa283 33326582 PMC8193555

[B148] Kang J. E. Lim M. M. Bateman R. J. Lee J. J. Smyth L. P. Cirrito J. R.. (2009). Amyloid-β dynamics are regulated by orexin and the sleep-wake cycle. Science 326, 1005–1007. 10.1126/science.1180962 19779148 PMC2789838

[B149] Karatsoreos I. N. Wang A. Sasanian J. Silver R. (2007). A role for androgens in regulating circadian behavior and the suprachiasmatic nucleus. Endocrinology 148, 5487–5495. 10.1210/en.2007-0775 17702841 PMC3281763

[B150] Kaur P. Jodhka P. K. Underwood W. A. Bowles C. A. de Fiebre N. C. de Fiebre C. M.. (2007). Progesterone increases brain-derived neurotrophic factor expression and protects against glutamate toxicity in a mitogen-activated protein kinase-and phosphoinositide-3 kinase-dependent manner in cerebral cortical explants. J. Neurosci. Res. 85, 2441–2449. 10.1002/jnr.21370 17549730 PMC2693123

[B151] Khan M. M. Dhandapani K. M. Zhang Q. G. Brann D. W. (2013). Estrogen regulation of spine density and excitatory synapses in rat prefrontal and somatosensory cerebral cortex. Steroids 78, 614–623. 10.1016/j.steroids.2012.12.005 23276632 PMC3640687

[B152] Kim P. Dufford A. J. Tribble R. C. (2018). Cortical thickness variation of the maternal brain in the first 6 months postpartum: associations with parental self-efficacy. Brain Struct. Funct. 223, 3267–3277. 10.1007/s00429-018-1688-z 29855765 PMC6358213

[B153] Kim P. Leckman J. F. Mayes L. C. Feldman R. Wang X. Swain J. E. (2010). The plasticity of human maternal brain: longitudinal changes in brain anatomy during the early postpartum period. Behav. Neurosci. 124, 695–700. 10.1037/a0020884 20939669 PMC4318549

[B154] Kinney J. W. Bemiller S. M. Murtishaw A. S. Leisgang A. M. Salazar A. M. Lamb B. T. (2018). Inflammation as a central mechanism in Alzheimer’s disease. Alzheimers Dement. (N Y) 4, 575–590. 10.1016/j.trci.2018.06.014 30406177 PMC6214864

[B155] Kinsley C. H. Trainer R. Stafisso-Sandoz G. Quadros P. Marcus L. K. Hearon C.. (2006). Motherhood and the hormones of pregnancy modify concentrations of hippocampal neuronal dendritic spines. Horm. Behav. 49, 131–142. 10.1016/j.yhbeh.2005.05.017 16005000

[B156] Konrad C. Engelien A. Schöning S. Zwitserlood P. Jansen A. Pletziger E.. (2008). The functional anatomy of semantic retrieval is influenced by gender, menstrual cycle and sex hormones. J. Neural Transm. (Vienna) 115, 1327–1337. 10.1007/s00702-008-0073-0 18548194 PMC2525845

[B157] Kramár E. A. Babayan A. H. Gall C. M. Lynch G. (2013). Estrogen promotes learning-related plasticity by modifying the synaptic cytoskeleton. Neuroscience 239, 3–16. 10.1016/j.neuroscience.2012.10.038 23103216 PMC4472431

[B158] Kravitz H. M. Ganz P. A. Bromberger J. Powell L. H. Sutton-Tyrrell K. Meyer P. M. (2003). Sleep difficulty in women at midlife: a community survey of sleep and the menopausal transition. Menopause 10, 19–28. 10.1097/00042192-200310010-00005 12544673

[B159] Kruijver F. P. Swaab D. F. (2002). Sex hormone receptors are present in the human suprachiasmatic nucleus. Neuroendocrinology 75, 296–305. 10.1159/000057339 12006783

[B160] Kumar P. Magon N. (2012). Hormones in pregnancy. Niger. Med. J. 53, 179–183. 10.4103/0300-1652.107549 23661874 PMC3640235

[B161] Lampio L. Polo-Kantola P. Himanen S. L. Kurki S. Huupponen E. Engblom J.. (2017). Sleep during menopausal transition: a 6-Year follow-up. Sleep 40:zsx090. 10.1093/sleep/zsx090 28525646

[B162] Lancel M. Faulhaber J. Holsboer F. Rupprecht R. (1996). Progesterone induces changes in sleep comparable to those of agonistic GABAA receptor modulators. Am. J. Physiol. 271, E763–E772. 10.1152/ajpendo.1996.271.4.E763 8897866

[B163] Lee J. Eklund E. E. Lambert-Messerlian G. Palomaki G. E. Butterfield K. Curran P.. (2017). Serum progesterone levels in pregnant women with obstructive sleep apnea: a case control study. J. Womens Health (Larchmt) 26, 259–265. 10.1089/jwh.2016.5917 28103130 PMC5361753

[B164] Lee K. A. Zaffke M. E. McEnany G. (2000). Parity and sleep patterns during and after pregnancy. Obstet. Gynecol. 95, 14–18. 10.1016/s0029-7844(99)00486-x 10636494

[B165] Li R. Singh M. (2014). Sex differences in cognitive impairment and Alzheimer’s disease. Front. Neuroendocrinol. 35, 385–403. 10.1016/j.yfrne.2014.01.002 24434111 PMC4087048

[B166] Li R. Shen Y. Yang L. B. Lue L. F. Finch C. Rogers J. (2000). Estrogen enhances uptake of amyloid β-protein by microglia derived from the human cortex. J. Neurochem. 75, 1447–1454. 10.1046/j.1471-4159.2000.0751447.x 10987824

[B167] Li X. Uemura K. Hashimoto T. Nasser-Ghodsi N. Arimon M. Lill C. M.. (2013). Neuronal activity and secreted amyloid β lead to altered amyloid β precursor protein and presenilin 1 interactions. Neurobiol. Dis. 50, 127–134. 10.1016/j.nbd.2012.10.002 23064434 PMC3534898

[B168] Lim A. S. Kowgier M. Yu L. Buchman A. S. Bennett D. A. (2013). Sleep fragmentation and the risk of incident Alzheimer’s disease and cognitive decline in older persons. Sleep 36, 1027–1032. 10.5665/sleep.2802 23814339 PMC3669060

[B169] Lima F. B. Leite C. M. Bethea C. L. Anselmo-Franci J. A. (2017). Progesterone increased β-endorphin innervation of the locus coeruleus, but ovarian steroids had no effect on noradrenergic neurodegeneration. Brain Res. 1663, 1–8. 10.1016/j.brainres.2017.03.008 28284896 PMC5425244

[B170] Linn M. C. Petersen A. C. (1985). Emergence and characterization of sex differences in spatial ability: a meta-analysis. Child Dev. 56, 1479–1498. 10.2307/1130467 4075870

[B171] Lisofsky N. Mårtensson J. Eckert A. Lindenberger U. Gallinat J. Kühn S. (2015). Hippocampal volume and functional connectivity changes during the female menstrual cycle. Neuroimage 118, 154–162. 10.1016/j.neuroimage.2015.06.012 26057590

[B172] Liu J. H. Lockwood C. J. Iams J. D. Greene M. F. (2013). “Endocrinology of pregnancy,” in Creasy and Resnik’s maternal-Fetal Medicine: Principles and Practice E-book, eds Resnik R. K. Greene R. Iams M. F. Lockwood J. D. Liu J. H. (Philadelphia, PA: Elsevier Health Sciences), 148–160.

[B173] Logan D. M. Hill K. R. Jones R. Holt-Lunstad J. Larson M. J. (2014). How do memory and attention change with pregnancy and childbirth? A controlled longitudinal examination of neuropsychological functioning in pregnant and postpartum women. J. Clin. Exp. Neuropsychol. 36, 528–539. 10.1080/13803395.2014.912614 24820853

[B174] Love G. Torrey N. McNamara I. Morgan M. Banks M. Hester N. W.. (2005). Maternal experience produces long-lasting behavioral modifications in the rat. Behav. Neurosci. 119, 1084–1096. 10.1037/0735-7044.119.4.1084 16187836

[B175] Luckey A. M. Robertson I. H. Lawlor B. Mohan A. Vanneste S. (2021). Sex differences in locus coeruleus: a heuristic approach that may explain the increased risk of Alzheimer’s disease in females. J. Alzheimers Dis. 83, 505–522. 10.3233/JAD-210404 34334399

[B176] Luders E. Gaser C. Gingnell M. Engman J. Sundström Poromaa I. Kurth F. (2021). Gray matter increases within subregions of the hippocampal complex after pregnancy. Brain Imaging Behav. 15, 2790–2794. 10.1007/s11682-021-00463-2 33881733

[B177] Lustig K. A. Stoakley E. M. MacDonald K. J. Geniole S. N. McCormick C. M. Cote K. A. (2017). Sex hormones play a role in vulnerability to sleep loss on emotion processing tasks. Neurobiol. Sleep Circadian Rhythms 5, 94–104. 10.1016/j.nbscr.2017.10.001 31236516 PMC6584637

[B178] Lynch M. A. (2004). Long-term potentiation and memory. Physiol. Rev. 84, 87–136. 10.1152/physrev.00014.2003 14715912

[B179] Macbeth A. H. Gautreaux C. Luine V. N. (2008). Pregnant rats show enhanced spatial memory, decreased anxiety and altered levels of monoaminergic neurotransmitters. Brain Res. 1241, 136–147. 10.1016/j.brainres.2008.09.006 18823955 PMC2652572

[B501] Macbeth A. H. Luine V. N. (2010). Changes in anxiety and cognition due to reproductive experience: a review of data from rodent and human mothers. Neurosci. Biobehav. Rev. 34, 452–467. 10.1016/j.neubiorev.2009.08.011 19761791

[B180] Majewska M. D. Harrison N. L. Schwartz R. D. Barker J. L. Paul S. M. (1986). Steroid hormone metabolites are barbiturate-like modulators of the GABA receptor. Science 232, 1004–1007. 10.1126/science.2422758 2422758

[B181] Maki P. M. (2013). The critical window hypothesis of hormone therapy and cognition: a scientific update on clinical studies. Menopause 20, 695–709. 10.1097/GME.0b013e3182960cf8 23715379 PMC3780981

[B182] Maki P. M. (2015). Verbal memory and menopause. Maturitas 82, 288–290. 10.1016/j.maturitas.2015.07.023 26433715

[B183] Maki P. M. Dennerstein L. Clark M. Guthrie J. LaMontagne P. Fornelli D.. (2011). Perimenopausal use of hormone therapy is associated with enhanced memory and hippocampal function later in life. Brain Res. 1379, 232–243. 10.1016/j.brainres.2010.11.030 21078303 PMC3046212

[B184] Maki P. M. Rich J. B. Rosenbaum R. S. (2002). Implicit memory varies across the menstrual cycle: estrogen effects in young women. Neuropsychologia 40, 518–529. 10.1016/s0028-3932(01)00126-9 11749982

[B185] Martin N. Lafortune M. Godbout J. Barakat M. Robillard R. Poirier G.. (2013). Topography of age-related changes in sleep spindles. Neurobiol. Aging 34, 468–476. 10.1016/j.neurobiolaging.2012.05.020 22809452

[B186] Martínez-García M. Paternina-Die M. Barba-Müller E. Martín de Blas D. Beumala L. Cortizo R.. (2021). Do pregnancy-induced brain changes reverse? The brain of a mother six years after parturition. Brain Sci. 11:168. 10.3390/brainsci11020168 33525512 PMC7912216

[B187] Matthews K. A. Lee L. Kravitz H. M. Joffe H. Neal-Perry G. Swanson L. M.. (2021). Influence of the menopausal transition on polysomnographic sleep characteristics: a longitudinal analysis. Sleep 44:zsab139. 10.1093/sleep/zsab139 34081126 PMC8598193

[B188] Mazure C. M. Swendsen J. (2016). Sex differences in Alzheimer’s disease and other dementias. Lancet. Neurol. 15, 451–452. 10.1016/S1474-4422(16)00067-3 26987699 PMC4864429

[B189] McDevitt E. A. Rokem A. Silver M. A. Mednick S. C. (2014). Sex differences in sleep-dependent perceptual learning. Vis. Res. 99, 172–179. 10.1016/j.visres.2013.10.009 24141074 PMC4704702

[B191] McEwen B. S. Milner T. A. (2017). Understanding the broad influence of sex hormones and sex differences in the brain. J. Neurosci. Res. 95, 24–39. 10.1002/jnr.23809 27870427 PMC5120618

[B192] McKinlay S. M. Brambilla D. J. Posner J. G. (1992). The normal menopause transition. Maturitas 14, 103–115. 10.1016/0378-5122(92)90003-m 1565019

[B193] Mendelsohn A. R. Larrick J. W. (2013). Sleep facilitates clearance of metabolites from the brain: glymphatic function in aging and neurodegenerative diseases. Rejuvenation Res. 16, 518–523. 10.1089/rej.2013.1530 24199995

[B194] Merlino G. Piani A. Gigli G. L. Cancelli I. Rinaldi A. Baroselli A.. (2010). Daytime sleepiness is associated with dementia and cognitive decline in older Italian adults: a population-based study. Sleep Med. 11, 372–377. 10.1016/j.sleep.2009.07.018 20219426

[B195] Mesiano S. (2001). Roles of estrogen and progesterone in human parturition. Front. Horm. Res. 27, 86–104. 10.1159/000061038 11450438

[B196] Mindell J. A. Cook R. A. Nikolovski J. (2015). Sleep patterns and sleep disturbances across pregnancy. Sleep Med. 16, 483–488. 10.1016/j.sleep.2014.12.006 25666847

[B197] Mirer A. G. Young T. Palta M. Benca R. M. Rasmuson A. Peppard P. E. (2017). Sleep-disordered breathing and the menopausal transition among participants in the sleep in midlife women study. Menopause 24, 157–162. 10.1097/GME.0000000000000744 27760083 PMC5266663

[B198] Moe K. E. Larsen L. H. Vitiello M. V. Prinz P. N. (2001). Estrogen replacement therapy moderates the sleep disruption associated with nocturnal blood sampling. Sleep 24, 886–894. 10.1093/sleep/24.8.886 11766158

[B199] Moline M. L. Broch L. Zak R. Gross V. (2003). Sleep in women across the life cycle from adulthood through menopause. Sleep Med. Rev. 7, 155–177. 10.1053/smrv.2001.0228 12628216

[B200] Mong J. A. Cusmano D. M. (2016). Sex differences in sleep: impact of biological sex and sex steroids. Philos. Trans. R. Soc. Lond. B Biol. Sci. 371:20150110. 10.1098/rstb.2015.0110 26833831 PMC4785896

[B201] Mong J. A. Devidze N. Frail D. E. O’Connor L. T. Samuel M. Choleris E.. (2003a). Estradiol differentially regulates lipocalin-type prostaglandin D synthase transcript levels in the rodent brain: evidence from high-density oligonucleotide arrays and *in situ* hybridization. Proc. Natl. Acad. Sci. U S A 100, 318–323. 10.1073/pnas.262663799 12518068 PMC140964

[B202] Mong J. A. Devidze N. Goodwillie A. Pfaff D. W. (2003b). Reduction of lipocalin-type prostaglandin D synthase in the preoptic area of female mice mimics estradiol effects on arousal and sex behavior. Proc. Natl. Acad. Sci. U S A 100, 15206–15211. 10.1073/pnas.2436540100 14638944 PMC299958

[B203] Montplaisir J. Lorrain J. Denesle R. Petit D. (2001). Sleep in menopause: differential effects of two forms of hormone replacement therapy. Menopause 8, 10–16. 10.1097/00042192-200101000-00004 11201509

[B204] Morong S. Hermsen B. de Vries N. (2014). Sleep-disordered breathing in pregnancy: a review of the physiology and potential role for positional therapy. Sleep Breath. 18, 31–37. 10.1007/s11325-013-0849-9 23591835

[B205] Morse J. K. Scheff S. W. DeKosky S. T. (1986). Gonadal steroids influence axon sprouting in the hippocampal dentate gyrus: a sexually dimorphic response. Exp. Neurol. 94, 649–658. 10.1016/0014-4886(86)90244-x 3780911

[B206] Mosconi L. Berti V. Dyke J. Schelbaum E. Jett S. Loughlin L.. (2021). Menopause impacts human brain structure, connectivity, energy metabolism and amyloid-β deposition. Sci. Rep. 11:10867. 10.1038/s41598-021-90084-y 34108509 PMC8190071

[B207] Moser D. Anderer P. Gruber G. Parapatics S. Loretz E. Boeck M.. (2009). Sleep classification according to AASM and Rechtschaffen & Kales: effects on sleep scoring parameters. Sleep 32, 139–149. 10.1093/sleep/32.2.139 19238800 PMC2635577

[B208] Nagata N. Urade Y. (2012). Sleep-wake regulation by prostaglandin D2 and adenosine. Brain Nerve 64, 621–628. 22647469

[B209] Nappi R. E. Sinforiani E. Mauri M. Bono G. Polatti F. Nappi G. (1999). Memory functioning at menopause: impact of age in ovariectomized women. Gynecol. Obstet. Invest. 47, 29–36. 10.1159/000010058 9852389

[B210] Nayak C. S. Anilkumar A. C. (2022). “EEG Normal Sleep,” in StatPearls [Internet]. Treasure Island, FL: StatPearls Publishing. 30725708

[B211] Nebel R. A. Aggarwal N. T. Barnes L. L. Gallagher A. Goldstein J. M. Kantarci K.. (2018). Understanding the impact of sex and gender in Alzheimer’s disease: a call to action. Alzheimers Dement. 14, 1171–1183. 10.1016/j.jalz.2018.04.008 29907423 PMC6400070

[B212] Nedergaard M. Goldman S. A. (2016). Brain drain. Sci. Am. 314, 44–49. 10.1038/scientificamerican0316-44 27066643 PMC5347443

[B213] Netzer N. C. Eliasson A. H. Strohl K. P. (2003). Women with sleep apnea have lower levels of sex hormones. Sleep Breath. 7, 25–29. 10.1007/s11325-003-0025-8 12712394

[B214] Nilsen J. Chen S. Irwin R. W. Iwamoto S. Brinton R. D. (2006). Estrogen protects neuronal cells from amyloid beta-induced apoptosis *via* regulation of mitochondrial proteins and function. BMC Neurosci. 7:74. 10.1186/1471-2202-7-74 17083736 PMC1636062

[B215] Ning K. Zhao L. Franklin M. Matloff W. Batta I. Arzouni N.. (2020). Parity is associated with cognitive function and brain age in both females and males. Sci. Rep. 10:6100. 10.1038/s41598-020-63014-7 32269255 PMC7142076

[B216] Nori R. Piccardi L. Maialetti A. Goro M. Rossetti A. Argento O.. (2018). No gender differences in egocentric and allocentric environmental transformation after compensating for male advantage by manipulating familiarity. Front. Neurosci. 12:204. 10.3389/fnins.2018.00204 29643763 PMC5882836

[B217] Oatridge A. Holdcroft A. Saeed N. Hajnal J. V. Puri B. K. Fusi L.. (2002). Change in brain size during and after pregnancy: study in healthy women and women with preeclampsia. Am. J. Neuroradiol. 23, 19–26. 11827871 PMC7975506

[B218] Pak V. M. Onen S. Bliwise D. L. Kutner N. G. Russell K. L. Onen F. (2020). Sleep disturbances in MCI and AD: neuroinflammation as a possible mediating pathway. Front. Aging Neurosci. 12, 69–69. 10.3389/fnagi.2020.00069 32457592 PMC7227443

[B219] Paul K. N. Dugovic C. Turek F. W. Laposky A. D. (2006). Diurnal sex differences in the sleep-wake cycle of mice are dependent on gonadal function. Sleep 29, 1211–1223. 10.1093/sleep/29.9.1211 17040009

[B220] Peter-Derex L. Yammine P. Bastuji H. Croisile B. (2015). Sleep and Alzheimer’s disease. Sleep Med. Rev. 19, 29–38. 10.1016/j.smrv.2014.03.007 24846773

[B221] Peterson A. Tom S. E. (2021). A lifecourse perspective on female sex-specific risk factors for later life cognition. Curr. Neurol. Neurosci. Rep. 21, 1–10. 10.1007/s11910-021-01133-y 34227023 PMC8449307

[B222] Phillips K. Silverman I. (1997). Differences in the relationship of menstrual cycle phase to spatial performance on two- and three-dimensional tasks. Horm. Behav. 32, 167–175. 10.1006/hbeh.1997.1418 9454667

[B223] Picard M. McEwen B. S. (2014). Mitochondria impact brain function and cognition. Proc. Natl. Acad. Sci. U S A 111, 7–8. 10.1073/pnas.1321881111 24367081 PMC3890847

[B224] Pien G. W. Pack A. I. Jackson N. Maislin G. Macones G. A. Schwab R. J.. (2014). Risk factors for sleep-disordered breathing in pregnancy. Thorax 69, 371–377. 10.1136/thoraxjnl-2012-202718 24262432 PMC6994201

[B225] Pike C. J. Carroll J. C. Rosario E. R. Barron A. M. (2009). Protective actions of sex steroid hormones in Alzheimer’s disease. Front. Neuroendocrinol. 30, 239–258. 10.1016/j.yfrne.2009.04.015 19427328 PMC2728624

[B226] Plamberger C. P. Van Wijk H. E. Kerschbaum H. Pletzer B. A. Gruber G. Oberascher K.. (2021). Impact of menstrual cycle phase and oral contraceptives on sleep and overnight memory consolidation. J. Sleep Res. 30:e13239. 10.1111/jsr.13239 33348471 PMC8365641

[B227] Pluchino N. Luisi M. Lenzi E. Centofanti M. Begliuomini S. Freschi L.. (2006). Progesterone and progestins: effects on brain, allopregnanolone and β-endorphin. J. Steroid Biochem. Mol. Biol. 102, 205–213. 10.1016/j.jsbmb.2006.09.023 17052903

[B228] Polo-Kantola P. (2007). Sleep and menopause. Women’s Health 3, 99–106. 10.2217/17455057.3.1.99 19803869

[B229] Postma A. Winkel J. Tuiten A. van Honk J. (1999). Sex differences and menstrual cycle effects in human spatial memory. Psychoneuroendocrinology 24, 175–192. 10.1016/s0306-4530(98)00073-0 10101726

[B230] Pozzo-Miller L. D. Inoue T. Murphy D. D. (1999). Estradiol increases spine density and NMDA-dependent Ca^2+^ transients in spines of CA1 pyramidal neurons from hippocampal slices. J. Neurophysiol. 81, 1404–1411. 10.1152/jn.1999.81.3.1404 10085365

[B231] Prince M. Bryce R. Albanese E. Wimo A. Ribeiro W. Ferri C. P. (2013). The global prevalence of dementia: a systematic review and metaanalysis. Alzheimers Dement. 9, 63–75.e2. 10.1016/j.jalz.2012.11.007 23305823

[B232] Prince T. M. Wimmer M. Choi J. Havekes R. Aton S. Abel T.. (2014). Sleep deprivation during a specific 3-hour time window post-training impairs hippocampal synaptic plasticity and memory. Neurobiol. Learn. Mem. 109, 122–130. 10.1016/j.nlm.2013.11.021 24380868 PMC3966473

[B233] Protopopescu X. Butler T. Pan H. Root J. Altemus M. Polanecsky M.. (2008). Hippocampal structural changes across the menstrual cycle. Hippocampus 18, 985–988. 10.1002/hipo.20468 18767068

[B234] Rahman A. Jackson H. Hristov H. Isaacson R. S. Saif N. Shetty T.. (2019). Sex and gender driven modifiers of Alzheimer’s: the role for estrogenic control across age, race, medical and lifestyle risks. Front. Aging Neurosci. 11:315. 10.3389/fnagi.2019.00315 31803046 PMC6872493

[B235] Rapp S. R. Espeland M. A. Shumaker S. A. Henderson V. W. Brunner R. L. Manson J. E.. (2003). Effect of estrogen plus progestin on global cognitive function in postmenopausal women: the women’s health initiative memory study: a randomized controlled trial. JAMA 289, 2663–2672. 10.1001/jama.289.20.2663 12771113

[B236] Rasch B. Born J. (2013). About sleep’s role in memory. Physiol. Rev. 93, 681–766. 10.1152/physrev.00032.2012 23589831 PMC3768102

[B237] Rechtschaffen A. Kales A. (1968). A Manual of Standardized Terminology, Techniques and Scoring System of Sleep Stages in Human Subbjects. Los Angeles: Brain Information Service/Brain Research Institute, University of California.

[B238] Reed B. G. Carr B. R. (2018). “The normal menstrual cycle and the control of ovulation,” in Endotext, ed Feingold K. R. (South Dartmouth, MA: MDText.com, Inc.).

[B500] Rettberg J. R. Yao J. Brinton R. D. (2014). Estrogen: a master regulator of bioenergetic systems in the brain and body. Front. Neuroendocrinol. 35, 8–30. 10.1016/j.yfrne.2013.08.001 23994581 PMC4024050

[B239] Rex C. S. Lauterborn J. C. Lin C. Y. Kramár E. A. Rogers G. A. Gall C. M.. (2006). Restoration of long-term potentiation in middle-aged hippocampus after induction of brain-derived neurotrophic factor. J. Neurophysiol. 96, 677–685. 10.1152/jn.00336.2006 16707719 PMC1554892

[B240] Ribeiro A. C. Pfaff D. W. Devidze N. (2009). Estradiol modulates behavioral arousal and induces changes in gene expression profiles in brain regions involved in the control of vigilance. Eur. J. Neurosci. 29, 795–801. 10.1111/j.1460-9568.2009.06620.x 19200066

[B241] Rocca W. A. Grossardt B. R. Shuster L. T. (2011). Oophorectomy, menopause, estrogen treatment and cognitive aging: clinical evidence for a window of opportunity. Brain Res. 1379, 188–198. 10.1016/j.brainres.2010.10.031 20965156 PMC3046246

[B242] Rolls A. Schori H. London A. Schwartz M. (2008). Decrease in hippocampal neurogenesis during pregnancy: a link to immunity. Mol. Psychiatry 13, 468–469. 10.1038/sj.mp.4002126 18421294

[B243] Romcy-Pereira R. Pavlides C. (2004). Distinct modulatory effects of sleep on the maintenance of hippocampal and medial prefrontal cortex LTP. Eur. J. Neurosci. 20, 3453–3462. 10.1111/j.1460-9568.2004.03808.x 15610178

[B244] Romeo R. D. McCarthy J. B. Wang A. Milner T. A. McEwen B. S. (2005). Sex differences in hippocampal estradiol-induced N-methyl-D-aspartic acid binding and ultrastructural localization of estrogen receptor-alpha. Neuroendocrinology 81, 391–399. 10.1159/000089557 16276117

[B245] Rosanova M. Ulrich D. (2005). Pattern-specific associative long-term potentiation induced by a sleep spindle-related spike train. J. Neurosci. 25, 9398–9405. 10.1523/JNEUROSCI.2149-05.2005 16221848 PMC6725710

[B246] Ross J. A. McGonigle P. Van Bockstaele E. J. (2015). Locus coeruleus, norepinephrine and Aβ peptides in Alzheimer’s disease. Neurobiol. Stress 2, 73–84. 10.1016/j.ynstr.2015.09.002 26618188 PMC4657149

[B247] Saadati H. Esmaeili-Mahani S. Esmaeilpour K. Nazeri M. Mazhari S. Sheibani V.. (2015). Exercise improves learning and memory impairments in sleep deprived female rats. Physiol. Behav. 138, 285–291. 10.1016/j.physbeh.2014.10.006 25447468

[B248] Saadati H. Sheibani V. Esmaeili-Mahani S. Darvishzadeh-Mahani F. Mazhari S. (2014a). Prior regular exercise reverses the decreased effects of sleep deprivation on brain-derived neurotrophic factor levels in the hippocampus of ovariectomized female rats. Regul. Pept. 194, 11–15. 10.1016/j.regpep.2014.11.004 25450575

[B249] Saadati H. Sheibani V. Esmaeili-Mahani S. Hajali V. Mazhari S. (2014b). Prior regular exercise prevents synaptic plasticity impairment in sleep deprived female rats. Brain Res. Bull. 108, 100–105. 10.1016/j.brainresbull.2014.09.009 25264158

[B250] Sabia S. Fayosse A. Dumurgier J. van Hees V. T. Paquet C. Sommerlad A.. (2021). Association of sleep duration in middle and old age with incidence of dementia. Nat. Commun. 12, 1–10. 10.1038/s41467-021-22354-2 33879784 PMC8058039

[B251] Saleh T. M. Connell B. J. (2003). Estrogen-induced autonomic effects are mediated by NMDA and GABAA receptors in the parabrachial nucleus. Brain Res. 973, 161–170. 10.1016/s0006-8993(03)02432-6 12738059

[B252] Saleh T. M. Saleh M. C. (2001). Inhibitory effect of 17β-estradiol in the parabrachial nucleus is mediated by GABA. Brain Res. 911, 116–124. 10.1016/s0006-8993(01)02699-3 11511378

[B253] Saletu B. (2003). Sleep, vigilance and cognition in postmenopausal women: Placebo-controlled studies with 2 mg estradiol valerate, with and without 3 mg dienogest. Climacteric 6, 37–45. 14669843

[B254] Sandstrom N. J. Williams C. L. (2001). Memory retention is modulated by acute estradiol and progesterone replacement. Behav. Neurosci. 115:384. 10.1037/0735-7044.115.2.384 11345963

[B255] Santoro N. Brown J. R. Adel T. Skurnick J. H. (1996). Characterization of reproductive hormonal dynamics in the perimenopause. J. Clin. Endocrinol. Metab. 81, 1495–1501. 10.1210/jcem.81.4.8636357 8636357

[B256] Saper C. B. Scammell T. E. Lu J. (2005). Hypothalamic regulation of sleep and circadian rhythms. Nature 437, 1257–1263. 10.1038/nature04284 16251950

[B257] Sattari N. McDevitt E. A. Panas D. Niknazar M. Ahmadi M. Naji M.. (2017). The effect of sex and menstrual phase on memory formation during a nap. Neurobiol. Learn. Mem. 145, 119–128. 10.1016/j.nlm.2017.09.007 28927742

[B258] Scammell T. Gerashchenko D. Urade Y. Onoe H. Saper C. Hayaishi O.. (1998). Activation of ventrolateral preoptic neurons by the somnogen prostaglandin D2. Proc. Natl. Acad. Sci. U S A 95, 7754–7759. 10.1073/pnas.95.13.7754 9636223 PMC22747

[B259] Schüssler P. Kluge M. Yassouridis A. Dresler M. Held K. Zihl J.. (2008). Progesterone reduces wakefulness in sleep EEG and has no effect on cognition in healthy postmenopausal women. Psychoneuroendocrinology 33, 1124–1131. 10.1016/j.psyneuen.2008.05.013 18676087

[B260] Shadyab A. H. Macera C. A. Shaffer R. A. Jain S. Gallo L. C. Gass M. L.. (2017). Ages at menarche and menopause and reproductive lifespan as predictors of exceptional longevity in women: the Women’s health initiative. Menopause 24:35. 10.1097/GME.0000000000000710 27465713 PMC5177476

[B261] Shah D. S. Prados J. Gamble J. De Lillo C. Gibson C. L. (2013). Sex differences in spatial memory using serial and search tasks. Behav. Brain Res. 257, 90–99. 10.1016/j.bbr.2013.09.027 24076150

[B262] Sharma S. Franco R. (2004). Sleep and its disorders in pregnancy. WMJ 103, 48–52. 10.5005/ijsm-1-2-72 15553565

[B263] Shokri-Kojori E. Wang G. J. Wiers C. E. Demiral S. B. Guo M. Kim S. W.. (2018). β-Amyloid accumulation in the human brain after one night of sleep deprivation. Proc. Natl. Acad. Sci. U S A 115, 4483–4488. 10.1073/pnas.1721694115 29632177 PMC5924922

[B264] Shughrue P. J. Lane M. V. Merchenthaler I. (1997). Comparative distribution of estrogen receptor-α and-β mRNA in the rat central nervous system. J. Comp. Neurol. 388, 507–525. 10.1002/(sici)1096-9861(19971201)388:4<507::aid-cne1>3.0.co;2-6 9388012

[B265] Shumaker S. A. Legault C. Kuller L. Rapp S. R. Thal L. Lane D. S.. (2004). Conjugated equine estrogens and incidence of probable dementia and mild cognitive impairment in postmenopausal women: Women’s health initiative memory study. JAMA 291, 2947–2958. 10.1001/jama.291.24.2947 15213206

[B266] Shumaker S. A. Legault C. Rapp S. R. Thal L. Wallace R. B. Ockene J. K.. (2003). Estrogen plus progestin and the incidence of dementia and mild cognitive impairment in postmenopausal women: the Women’s health initiative memory study: a randomized controlled trial. JAMA 289, 2651–2662. 10.1001/jama.289.20.2651 12771112

[B267] Siddiqui A. N. Siddiqui N. Khan R. A. Kalam A. Jabir N. R. Kamal M. A.. (2016). Neuroprotective role of steroidal sex hormones: an overview. CNS Neurosci. Ther. 22, 342–350. 10.1111/cns.12538 27012165 PMC6492877

[B268] Silvestri R. Aricò I. (2019). Sleep disorders in pregnancy. Sleep Sci. 12, 232–239. 10.5935/1984-0063.20190098 31890101 PMC6932848

[B269] Sliwinski A. Monnet F. P. Schumacher M. Morin-Surun M. P. (2004). Pregnenolone sulfate enhances long-term potentiation in CA1 in rat hippocampus slices through the modulation of N-methyl-D-aspartate receptors. J. Neurosci. Res. 78, 691–701. 10.1002/jnr.20332 15505794

[B270] Smith R. Smith J. I. Shen X. Engel P. J. Bowman M. E. McGrath S. A.. (2009). Patterns of plasma corticotropin-releasing hormone, progesterone, estradiol and estriol change and the onset of human labor. J. Clin. Endocrinol. Metab. 94, 2066–2074. 10.1210/jc.2008-2257 19258402

[B271] Snyder H. M. Asthana S. Bain L. Brinton R. Craft S. Dubal D. B.. (2016). Sex biology contributions to vulnerability to Alzheimer’s disease: a think tank convened by the Women’s Alzheimer’s Research Initiative. Alzheimer’s Dement. 12, 1186–1196. 10.1016/j.jalz.2016.08.004 27692800 PMC10341380

[B272] Soares C. N. (2005). Insomnia in women: an overlooked epidemic? Arch. Womens Ment. Health 8, 205–213. 10.1007/s00737-005-0100-1 16195781

[B273] Spira A. P. Gamaldo A. A. An Y. Wu M. N. Simonsick E. M. Bilgel M.. (2013). Self-reported sleep and β-amyloid deposition in community-dwelling older adults. JAMA Neurol. 70, 1537–1543. 10.1001/jamaneurol.2013.4258 24145859 PMC3918480

[B274] Sprecher K. E. Bendlin B. B. Racine A. M. Okonkwo O. C. Christian B. T. Koscik R. L.. (2015). Amyloid burden is associated with self-reported sleep in nondemented late middle-aged adults. Neurobiol. Aging 36, 2568–2576. 10.1016/j.neurobiolaging.2015.05.004 26059712 PMC4523445

[B276] Squire L. R. Genzel L. Wixted J. T. Morris R. G. (2015). Memory consolidation. Cold Spring Harb. Perspect. Biol. 7:a021766. 10.1101/cshperspect.a021766 26238360 PMC4526749

[B275] Squire L. R. Zola S. M. (1996). Structure and function of declarative and nondeclarative memory systems. Proc. Natl. Acad. Sci. U S A 93, 13515–13522. 10.1073/pnas.93.24.13515 8942965 PMC33639

[B277] Stahl M. L. Orr W. C. Males J. L. (1985). Progesterone levels and sleep-related breathing during menstrual cycles of normal women. Sleep 8, 227–230. 10.1093/sleep/8.3.227 4048738

[B278] Stickgold R. (2005). Sleep-dependent memory consolidation. Nature 437, 1272–1278. 10.1038/nature04286 16251952

[B279] Sundström Poromaa I. Gingnell M. (2014). Menstrual cycle influence on cognitive function and emotion processing—from a reproductive perspective. Front. Neurosci. 8:380. 10.3389/fnins.2014.00380 25505380 PMC4241821

[B280] Tabuchi M. Lone S. R. Liu S. Liu Q. Zhang J. Spira A. P.. (2015). Sleep interacts with aβ to modulate intrinsic neuronal excitability. Curr. Biol. 25, 702–712. 10.1016/j.cub.2015.01.016 25754641 PMC4366315

[B281] Tanapat P. Hastings N. B. Gould E. (2005). Ovarian steroids influence cell proliferation in the dentate gyrus of the adult female rat in a dose-and time-dependent manner. J. Comp. Neurol. 481, 252–265. 10.1002/cne.20385 15593136

[B282] Tatsumi I. F. Watanabe M. (2009). “Verbal memory,” in Encyclopedia of Neuroscience, eds Binder M. D. Hirokawa N. Windhorst U. (Berlin, Heidelberg: Springer).

[B283] Terán-Pérez G. Arana-Lechuga Y. Esqueda-León E. Santana-Miranda R. Rojas-Zamorano J. A. Velázquez Moctezuma J.. (2012). Steroid hormones and sleep regulation. Mini Rev. Med. Chem. 12, 1040–1048. 10.2174/138955712802762167 23092405

[B284] Toffoletto S. Lanzenberger R. Gingnell M. Sundström-Poromaa I. Comasco E. (2014). Emotional and cognitive functional imaging of estrogen and progesterone effects in the female human brain: a systematic review. Psychoneuroendocrinology 50, 28–52. 10.1016/j.psyneuen.2014.07.025 25222701

[B285] Tononi G. Cirelli C. (2006). Sleep function and synaptic homeostasis. Sleep Med. Rev. 10, 49–62. 10.1016/j.smrv.2005.05.002 16376591

[B286] Tulchinsky D. Hobel C. J. Yeager E. Marshall J. R. (1972). Plasma estrone, estradiol, estriol, progesterone and 17-hydroxyprogesterone in human pregnancy: I. Normal pregnancy. Am. J. Obstet. Gynecol. 112, 1095–1100. 10.1016/0002-9378(72)90185-8 5025870

[B287] Ujma P. P. Konrad B. N. Genzel L. Bleifuss A. Simor P. Pótári A.. (2014). Sleep spindles and intelligence: evidence for a sexual dimorphism. J. Neurosci. 34, 16358–16368. 10.1523/JNEUROSCI.1857-14.2014 25471574 PMC6608484

[B288] Venkata C. Venkateshiah S. B. (2009). Sleep-disordered breathing during pregnancy. J. Am. Board Fam. Med. 22, 158–168. 10.3122/jabfm.2009.02.080057 19264939

[B289] Vest R. S. Pike C. J. (2013). Gender, sex steroid hormones and Alzheimer’s disease. Horm. Behav. 63, 301–307. 10.1016/j.yhbeh.2012.04.006 22554955 PMC3413783

[B290] Vida B. Hrabovszky E. Kalamatianos T. Coen C. W. Liposits Z. Kalló I.. (2008). Oestrogen receptor α and β immunoreactive cells in the suprachiasmatic nucleus of mice: distribution, sex differences and regulation by gonadal hormones. J. Neuroendocrinol. 20, 1270–1277. 10.1111/j.1365-2826.2008.01787.x 18752649

[B291] Wang M. (2011). Neurosteroids and GABA-A receptor function. Front. Neuroendocr. sci. 2, 1–23. 10.3389/fendo.2011.00044 22654809 PMC3356040

[B292] Wang W. Le A. A. Hou B. Lauterborn J. C. Cox C. D. Levin E. R.. (2018). Memory-related synaptic plasticity is sexually dimorphic in rodent hippocampus. J. Neurosci. 38, 7935–7951. 10.1523/JNEUROSCI.0801-18.2018 30209204 PMC6136152

[B293] Waters E. M. Yildirim M. Janssen W. G. Lou W. W. McEwen B. S. Morrison J. H.. (2011). Estrogen and aging affect the synaptic distribution of estrogen receptor beta-immunoreactivity in the CA1 region of female rat hippocampus. Brain Res. 1379, 86–97. 10.1016/j.brainres.2010.09.069 20875808 PMC3046233

[B294] Weber M. T. Mapstone M. Staskiewicz J. Maki P. M. (2012). Reconciling subjective memory complaints with objective memory performance in the menopausal transition. Menopause 19, 735–741. 10.1097/gme.0b013e318241fd22 22415562 PMC3773730

[B295] Wei Y. Krishnan G. P. Komarov M. Bazhenov M. (2018). Differential roles of sleep spindles and sleep slow oscillations in memory. PLoS Comput. Biol. 22, 8691–8704. 10.1371/journal.pcbi.1006322 29985966 PMC6053241

[B296] Weiss E. M. Kemmler G. Deisenhammer E. A. Fleischhacker W. W. Delazer M. (2003). Sex differences in cognitive functions. Personal. Indiv. Differ. 35, 863–875. 10.1016/S0191-8869(02)00288-X

[B297] Whitmer R. A. Quesenberry C. P. Zhou J. Yaffe K. (2011). Timing of hormone therapy and dementia: The critical window theory revisited. Ann. Neurol. 69, 163–169. 10.1002/ana.22239 21280086 PMC3058824

[B298] Wilson D. L. Barnes M. Ellett L. Permezel M. Jackson M. Crowe S. F.. (2011a). Compromised verbal episodic memory with intact visual and procedural memory during pregnancy. J. Clin. Exp. Neuropsychol. 33, 680–691. 10.1080/13803395.2010.550604 21409694

[B299] Wilson D. L. Barnes M. Ellett L. Permezel M. Jackson M. Crowe S. F.. (2011b). Decreased sleep efficiency, increased wake after sleep onset and increased cortical arousals in late pregnancy. Aust. N Z J. Obstet. Gynaecol. 51, 38–46. 10.1111/j.1479-828X.2010.01252.x 21299507

[B300] Wilson D. L. Barnes M. Ellett L. Permezel M. Jackson M. Crowe S. F.. (2013). Reduced verbal memory retention is unrelated to sleep disturbance during pregnancy. Aust. Psychol. 48, 196–208. 10.1111/j.1742-9544.2012.00076.x

[B301] Winer J. R. Deters K. D. Kennedy G. Jin M. Goldstein-Piekarski A. Poston K. L.. (2021). Association of short and long sleep duration with amyloid-β burden and cognition in aging. JAMA Neurologyneurol. 78, 1187–1196. 10.1001/jamaneurol.2021.2876 34459862 PMC8406215

[B302] Winer J. R. Mander B. A. Kumar S. Reed M. Baker S. L. Jagust W. J.. (2020). Sleep disturbance forecasts β-amyloid accumulation across subsequent years. Curr. Biol. 30, 4291–4298. 10.1016/j.cub.2020.08.017 32888482 PMC7642104

[B304] Woolley C. S. Gould E. Frankfurt M. McEwen B. S. (1990). Naturally occurring fluctuation in dendritic spine density on adult hippocampal pyramidal neurons. J. Neurosci. 10, 4035–4039. 10.1523/JNEUROSCI.10-12-04035.1990 2269895 PMC6570039

[B303] Woolley C. S. McEwen B. S. (1993). Roles of estradiol and progesterone in regulation of hippocampal dendritic spine density during the estrous cycle in the rat. J. Comp. Neurol. 336, 293–306. 10.1002/cne.903360210 8245220

[B305] Workman J. L. Barha C. K. Galea L. A. (2012). Endocrine substrates of cognitive and affective changes during pregnancy and postpartum. Behav. Neurosci. 126, 54–72. 10.1037/a0025538 21967374

[B306] World Health Organization (2021). Dementia. World Health Organization. Available online at: http://www.who.int/news-room/fact-sheets/detail/dementia. Accessed September 6, 2021.

[B307] World Health Statistics (2017). Mental Health of Older Adults. Available online at http://www.who.int/mediacentre/factsheets/fs381/en/.

[B308] Xie L. Kang H. Xu Q. Chen M. J. Liao Y. Thiyagarajan M.. (2013). Sleep drives metabolite clearance from the adult brain. Science 342, 373–377. 10.1126/science.1241224 24136970 PMC3880190

[B309] Xu Q. Lang C. P. (2014). Examining the relationship between subjective sleep disturbance and menopause: a systematic review and meta-analysis. Menopause 21, 1301–1318. 10.1097/GME.0000000000000240 24800878

[B310] Yaffe K. Laffan A. M. Harrison S. L. Redline S. Spira A. P. Ensrud K. E.. (2011). Sleep-disordered breathing, hypoxia and risk of mild cognitive impairment and dementia in older women. JAMA 306, 613–619. 10.1001/jama.2011.1115 21828324 PMC3600944

[B311] Yankova M. Hart S. A. Woolley C. S. (2001). Estrogen increases synaptic connectivity between single presynaptic inputs and multiple postsynaptic CA1 pyramidal cells: a serial electron-microscopic study. Proc. Natl. Acad. Sci. U S A 98, 3525–3530. 10.1073/pnas.051624598 11248111 PMC30686

[B312] Young T. Rabago D. Zgierska A. Austin D. Laurel F. (2003). Objective and subjective sleep quality in premenopausal, perimenopausal and postmenopausal women in the wisconsin sleep cohort study. Sleep 26, 667–672. 10.1093/sleep/26.6.667 14572118

[B313] Zárate S. Stevnsner T. Gredilla R. (2017). Role of estrogen and other sex hormones in brain aging. Neuroprotection and DNA repair. Front. Aging Neurosci. 9:430. 10.3389/fnagi.2017.00430 29311911 PMC5743731

[B314] Zeydan B. Lowe V. J. Tosakulwong N. Lesnick T. G. Senjem M. L. Jack C. R.. (2021). Sleep quality and cortical amyloid-β deposition in postmenopausal women of the Kronos early estrogen prevention study. Neuroreport 32, 326–331. 10.1097/WNR.0000000000001592 33470769 PMC7878341

[B316] Zhang X. Cao D. Sun J. Shao D. Sun Y. Cao F.. (2021). Sleep heterogeneity in the third trimester of pregnancy: correlations with depression, memory impairment and fatigue. Psychiatry Res. 303:114075. 10.1016/j.psychres.2021.114075 34198213

[B315] Zhang B. Wing Y. K. (2006). Sex differences in insomnia: A meta-analysis. Sleep 29, 85–93. 10.1093/sleep/29.1.85 16453985

[B317] Zheng H. Harlow S. D. Kravitz H. M. Bromberger J. Buysse D. J. Matthews K. A.. (2015). Actigraphy-defined measures of sleep and movement across the menstrual cycle in midlife menstruating women: study of Women’s health across the nation sleep study. Menopause 22, 66–74. 10.1097/GME.0000000000000249 24845393 PMC4237700

